# Deeplasmid: deep learning accurately separates plasmids from bacterial chromosomes

**DOI:** 10.1093/nar/gkab1115

**Published:** 2021-12-06

**Authors:** William B Andreopoulos, Alexander M Geller, Miriam Lucke, Jan Balewski, Alicia Clum, Natalia N Ivanova, Asaf Levy

**Affiliations:** Joint Genome Institute, US Department of Energy, LBNL Berkeley, CA, USA; Department of Computer Science, San Jose State University, CA, USA; Department of Plant Pathology and Microbiology, The Institute of Environmental Science, The Robert H. Smith Faculty of Agriculture, Food, and Environment, The Hebrew University of Jerusalem, Rehovot, Israel; Department of Plant Pathology and Microbiology, The Institute of Environmental Science, The Robert H. Smith Faculty of Agriculture, Food, and Environment, The Hebrew University of Jerusalem, Rehovot, Israel; National Energy Research Scientific Computing Center (NERSC), Berkeley, CA, USA; Joint Genome Institute, US Department of Energy, LBNL Berkeley, CA, USA; Joint Genome Institute, US Department of Energy, LBNL Berkeley, CA, USA; Department of Plant Pathology and Microbiology, The Institute of Environmental Science, The Robert H. Smith Faculty of Agriculture, Food, and Environment, The Hebrew University of Jerusalem, Rehovot, Israel

## Abstract

Plasmids are mobile genetic elements that play a key role in microbial ecology and evolution by mediating horizontal transfer of important genes, such as antimicrobial resistance genes. Many microbial genomes have been sequenced by short read sequencers and have resulted in a mix of contigs that derive from plasmids or chromosomes. New tools that accurately identify plasmids are needed to elucidate new plasmid-borne genes of high biological importance. We have developed Deeplasmid, a deep learning tool for distinguishing plasmids from bacterial chromosomes based on the DNA sequence and its encoded biological data. It requires as input only assembled sequences generated by any sequencing platform and assembly algorithm and its runtime scales linearly with the number of assembled sequences. Deeplasmid achieves an AUC–ROC of over 89%, and it was more accurate than five other plasmid classification methods. Finally, as a proof of concept, we used Deeplasmid to predict new plasmids in the fish pathogen *Yersinia ruckeri* ATCC 29473 that has no annotated plasmids. Deeplasmid predicted with high reliability that a long assembled contig is part of a plasmid. Using long read sequencing we indeed validated the existence of a 102 kb long plasmid, demonstrating Deeplasmid's ability to detect novel plasmids.

## INTRODUCTION

Plasmids are ubiquitous extrachromosomal elements capable of semi-autonomous replication and transmission between microbial host cells. Typically, bacterial plasmids are small (<80 kb) circular replicons. Natural plasmids often carry a cargo of ‘accessory genes’ that confer beneficial traits to the microbial host, such as antibacterial resistance (1,2), bacteriophage defense (3,4), heavy metal tolerance (5), virulence (6,7) or unique catabolic pathways (8), thereby improving bacterial adaptation to dynamic environments. Some plasmids carry toxins and thereby constitute a serious threat to human health (9). Finally, plasmids are involved in plant-microbe interactions; for instance, the nodulation plasmids of rhizobia guide the symbiosis of bacteria with plants (10). Plasmid transmission by conjugation provides an efficient mechanism of horizontal gene transfer and facilitates the spread of accessory genes in bacterial populations and communities. Therefore, the studies of plasmid genetics, evolution, and dynamics in bacterial populations have many wide-reaching practical applications, such as clinical management of antibiotic resistance (2), development of industrial strains of bacteria for bioremediation (3) and biofertilization (4). In addition, identification of new plasmids may guide the discovery of novel antibiotic resistance genes, toxins, and genes that directly shape host-microbe interactions, and these plasmids can be used as new tools for efficient gene cloning and exogenous protein expression.

Advances in genomic sequencing technologies have enabled high-throughput sequencing of genomes of microbial isolates and environmental populations (through metagenome sequencing), including their respective plasmidomes—the total collection of encoded plasmids (5). Identification and classification of plasmid sequences in this treasure trove of genomic and metagenomic data can provide a unique opportunity to study the mechanisms of plasmid persistence, transmission, and host specificity, as well as the flow and evolution of plasmidic accessory genes. However, *in silico* identification of plasmid contigs in whole-genome shotgun sequences (WGS) is challenging. The challenge derives from poor genome assembly that leads to numerous plasmid-size contigs that are difficult to characterize as derived from plasmid or chromosomes. In addition, there is a limited number of high-quality, completely sequenced reference plasmids that can be compared to while annotating new genomes (6). Sequences from plasmids occasionally integrate into chromosomes, making it difficult to computationally characterize contigs from these chromosomes as plasmids or chromosomes. Further, sometimes plasmid genes have features resembling those of essential chromosomal genes (11).

A variety of *in silico* methods assisting with separation of plasmid sequences from chromosomal contigs have been developed. Some of them target a subset of plasmids mostly of clinical relevance, such as PlasmidFinder/pMLST (12) for detection and typing of plasmids from *Enterobacteriaceae* and selected Gram-positive strains. Other tools, such as PLACNET (13) and the Kraken classifier with a custom database (14) rely on a combination of reference genomes and manual curation to restructure an assembly graph and to separate putative plasmid contigs from those of chromosomal origin. PlasmidSPAdes (15), cBAR (16), PlasFlow (17), Recycler (18) and PlasmidSeeker (19) are fully automated and perform identification of putative plasmid contigs in genome assemblies by analyzing the topology and read coverage of an assembly graph (Recycler and plasmidSPAdes) or DNA composition of assembled contigs (cBar and PlasFlow) or unassembled DNA (PlasmidSeeker). Recycler works on paired-end reads and detects circular plasmids by leveraging assembly graphs from conventional assembly tools to assemble circular sequences likely to be plasmids (18). HyAsP starts from raw reads and combines read depth with GC content, as well as reference-based occurrences of known plasmid genes in the assembly (20). An assessment of methods that assemble plasmids from short reads (21) concluded that their accuracy is reliant on a difference in the coverage of plasmids and chromosomes; for some assemblies they demonstrated close to a 90% precision in plasmid finding with just 55% recall, whereas for assemblies with 80% recall generally the false positive rate increases by 20% (21). Moreover, most of these tools were not tested for their ability to detect novel plasmids that are experimentally validated following the computational prediction.

Existing tools have limitations due to their reliance on the circularity of the topology, bias towards certain taxonomies used in training (e.g. PlasmidFinder, PLACNET, Kraken) and coverage of a de Bruijn assembly graph constructed from k-mers found in reads (e.g. Recycler and plasmidSPAdes). The software packages cBAR (16), PlasFlow (17), mlplasmids (22), Platon (23) and PlasmidVerify (24) satisfy the above criteria, since they utilize only two types of data: assembled sequences themselves (PlasFlow) and features extracted from assembled sequences (cBar, mlplasmids, Platon, PlasmidVerify). PlasFlow relies on a deep neural network to find hidden structures encoded in the assembled sequences, while cBar finds plasmids by applying self-organizing maps to the extracted features in the form of pentamer profiles of contigs and scaffolds. Various methods also differ in the way their models are trained: PlasFlow was developed as a tool for finding plasmids in metagenome data and is pre-trained on sequence fragments of up to 10 kb long, since metagenome assemblies are typically very fragmented. In contrast, the cBAR and mlplasmids models are based on the pentanucleotide profiles of full-length sequences of known plasmids and chromosomes. cBAR and PlasFlow have demonstrated superior performance in comparison to other methods of plasmid identification (17).

Our goal was to develop a tool for post-assembly identification of complete plasmids and plasmid-derived contigs, which (i) has high accuracy, (ii) is not biased towards the sequences of certain topology or taxonomic origin and (iii) is able to run on genome assemblies from either short-read or long-read sequencing technologies without assembly graph or coverage information. We also confirmed that inclusion of genes, protein domains, and other functional features in addition to DNA sequence composition is helpful for contig and scaffold classification. We present a new Deep Learning (DL)-based method, Deeplasmid, for identification of plasmid contigs and scaffolds in WGS assemblies of microbial isolate genomes, which achieves an AUC-ROC of 98.8% on a sixfold cross-validation. Our method relies on a combination of assembled sequences and extracted features, including GC content (25), oligonucleotide composition, hits to plasmid- or chromosome-specific genes and protein domains, as well as gene density within the contig. Since it does not require raw read data, assembly graph or coverage information, it can be applied to assembled WGS data, including shotgun metagenomic data, generated by any sequencing platform and assembly algorithm. We describe our Deeplasmid model, the training and testing methodology, and show that it is capable of automated detection of plasmid sequences with over 84% accuracy. Deeplasmid surpasses the accuracy of other tools largely due to its use of discriminating gene and protein features. We compared our trained model on large plasmid-containing microbial test datasets against the alternative tools cBAR, PlasFlow, PlasmidVerify, Platon and mlplasmids. Deeplasmid achieved higher accuracy than the other tools. Finally, we applied Deeplasmid and predicted a novel plasmid in the fish pathogen *Yersinia ruckeri* ATCC 29473. We then performed a re-sequencing experiment to validate that the new plasmid indeed exists as a separate replication unit. This led to discovery of a new plasmid in this pathogenic strain.

## MATERIALS AND METHODS

### Training dataset assembly

We prepared the labeled dataset based on three sources. As negative instances we used 40 000 sequences from 930 different genera from the RefSeq.bacteria dataset (26), from which plasmid and any mitochondrial or chloroplast sequences were removed based on their fasta header names. The training included also 3094 Archaeal chromosomal sequences from 45 genera, which are found in RefSeq.archaea. As positive instances we used the ACLAME dataset (27), which contains 1056 fully-sequenced plasmids that were manually curated by experts, as well as the PLSDB database, which contains 27 939 fully-sequenced plasmids. ACLAME and PLSDB have higher-quality curation than refseq.plasmids since some of the NCBI records tagged as plasmids are mislabeled as chromosomal sequences and many entries do not represent complete records or contain sequence fragments of unknown origin (28,29). From ACLAME we discarded 39 sequences (3.69%) and from PLSDB we discarded 1469 sequences (5.2%) that were shorter than 1 kb or longer than 330 kb because the scaffolds and contigs longer than 330 kb are usually chromosomes, megaplasmids or chromids (genetic elements with plasmid-type replication systems, but carrying some indispensable genes (11)). We did not deal with the last two classes as they are special cases. Data were shuffled before training.

### Input format

A single training data element consists of the label and two input words: *x*_seq_—a 300 bp contiguous subsequence sampled randomly from the full original scaffold sequence and *x*_f_—a vector containing 16 features and 1538 Pfam hits extracted from the full sequence, as described in Table 1. In order to ensure feature values like gene count, gene coding percentage, or sequence length are meaningful, the features are computed on the entire scaffold, and the values are copied into the *x*_f_ feature vectors for all 300 bp sequences subsampled from the scaffold. The number (*m*) of 300 bp subsequences sampled from each scaffold is proportional to the square root of the scaffold length. The number of samples per scaffold was chosen according to *m* = 10 + sqrt(seq_len/20) to ensure a fair representation of smaller and larger scaffolds, such that longer scaffolds do not overwhelm the training step. Each sample is a different *x*_seq_ associated with the feature vector *x*_f_ from the originating sequence. *x*_seq_ is one-hot encoded in four nucleotide bases. Namely, it is transformed into a binary array of size 300 × 4. Sequences with any ‘*N*’-bases are removed. The values of *x*_f_ were normalized to be bound within [–1,1].

**Table 1.** Definition of features per sequence

Name	Definition	Type
gc_content	GC content of contig	Float [0–1]
A(C/G/T)_longest_homopolymer	Length of longest homopolymer	Integer
A(C/G/T)_total_homopolymer	Total number of homopolymers of length >5	Integer
hit_chromosome_proteins	Hit to chromosome proteins	Boolean 0/1
hit_plasmid_proteins	Hit to plasmid proteins	Boolean 0/1
hit_plasmid_ORIs	Hit to plasmid ORI	Boolean 0/1
gene_count	Number of genes in scaffold	Integer
gene_percent	Coding percent of scaffold	Float [0–1]
polypeptide_aa_avg_len	Average length of amino acid sequence	Integer
len_sequence	Scaffold seq length	Integer
pfam_vector	1538 boolean (0 or 1) Pfam hits	Vector

### Input feature selection

We initially explored the predictive power of several extracted features in conjunction with existing Machine Learning tools. The particular choice of *x*_f_ variables shown in Table 1 was based on an initial sensitivity analysis with the Gradient Boosting Classifier, a classic ML method that produces a prediction model in the form of a mixture of decision trees. Moreover, we confirmed the relative importance of a feature by training our tool and running predictions with null values for the feature (namely, excluding the feature) and checking the impact on the error rate (as discussed in the Supplementary Info).

We additionally included in *x*_f_ plasmid-specific and chromosome-specific features. These features are boolean (0 or 1) and indicate whether any hit is found to these sets of plasmid or chromosome-specific sequences. Overall, we used thousands of plasmid-specific and chromosome-specific proteins, Pfam domains, and DNA motifs as features in the classification process, including sequences manually curated from the literature. Here are the features that were used:

- Plasmid-specific DNA motifs: these are the origins of replication of known plasmids (30). Sixteen origin of replication sequences were used.
- Plasmid-specific proteins: these are taken from 2826 known plasmids listed on 2019 in the European Nucleotide Archive: https://www.ebi.ac.uk/genomes/plasmid.html, after removing any plasmids not found in Proteobacteria, Firmicutes, Bacteroidetes, or Actinobacteria using NCBI batch entrez function. We only kept plasmids from these four phyla as these are the most commonly sequenced and studied bacterial phyla and as a result most contigs that will be classified by our tool belong to these phyla. Some plasmidic proteins were extracted from publications (31–40) (Supplementary Table S1). The final list included 136 638, 24 607, 1163 and 15 449 plasmidic genes from Proteobacteria, Firmicutes, Bacteroidetes and Actinobacteria, respectively.
- Chromosome-specific proteins: these are based on COGs of genes that are usually carried on chromosomes. The 61 COGs used for making this list are based on chromosomal housekeeping genes that are unclonable in high copy plasmids (41,42). They appear in Supplementary Table S1. To reduce sequence redundancy the chromosomal and plasmid proteins were clustered by 90% identity using cd-hit with otherwise default parameters giving a representative sequence from each cluster (43).
- Chromosome and plasmid-specific Pfam domains (Supplementary Table S2): bacterial scaffolds from the IMG database that were >1 Mb in size were considered to be chromosomal in nature. Genes from these scaffolds annotated with Pfams were collected. DNA sequence accessions of plasmids from PLSDB were cross-referenced with their corresponding protein entries on NCBI, and those with Pfam annotations were collected. Equal numbers of Pfams were sampled randomly from the list of chromosomal and plasmid Pfams (70 000 each). We calculated the frequencies of each Pfam in either chromosomal or plasmid Pfams to normalize them, and then compared the ratio of frequencies. This resulted in a sorted list of Pfams that were enriched in plasmids at one end of the spectrum, and Pfams enriched in chromosomes at the other end of the spectrum. We shortened this list by keeping 800 plasmid-specific Pfams that occurred 10 times more frequently in plasmids than in chromosomes, and 738 chromosome-specific Pfams occurring 20 times more frequently in chromosomes than in plasmids. The result was a feature list of the 1538 strongest marker Pfam domains that are checked for presence or absence in a scaffold.

### Output Format

A neural network is a function

(1) $$\begin{equation*}{{F}}\left( {{{{x}}_{{\rm{seq}}}}{\rm{,}}{{{x}}_{\rm{f}}}{\rm{|\theta }}} \right){{ = y}}.\end{equation*}$$

that accepts sequences of nucleotides *x*_seq_ and the feature vector *x*_f_. The function *F* also depends implicitly on the DL model parameters θ, which are determined during the training process. The output of the network, Deeplasmid score *y*, is computed using the softmax function, which ensures that *y* satisfies *y* ∈ [0, 1]. By convention, the higher the score is for the sequence, the more likely it is to be a true plasmid.

### Model training

The model was trained with a binary cross-entropy loss function (44) and Adam optimizer (45). We performed supervised learning on the balanced set of 6 × 10^5^ data points with a batch size of 200. The initial learning rate was set to 0.001. Typically we sampled 50–100 300 bp long sequences per scaffold.

**Figure 1.**
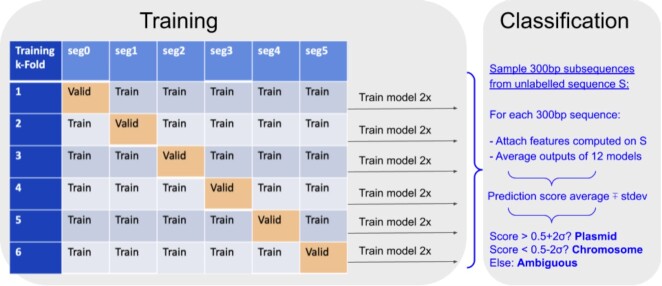
Deeplasmid training, validation and testing. The plasmid and chromosome dataset was split into six segments, of which five were used in training a model. The sixth segment was used for validation of the trained model. We repeated over the training twice to derive 12 different models. Using 12 models allows reducing the effects of random variance in the predictions.

**Figure 2.**
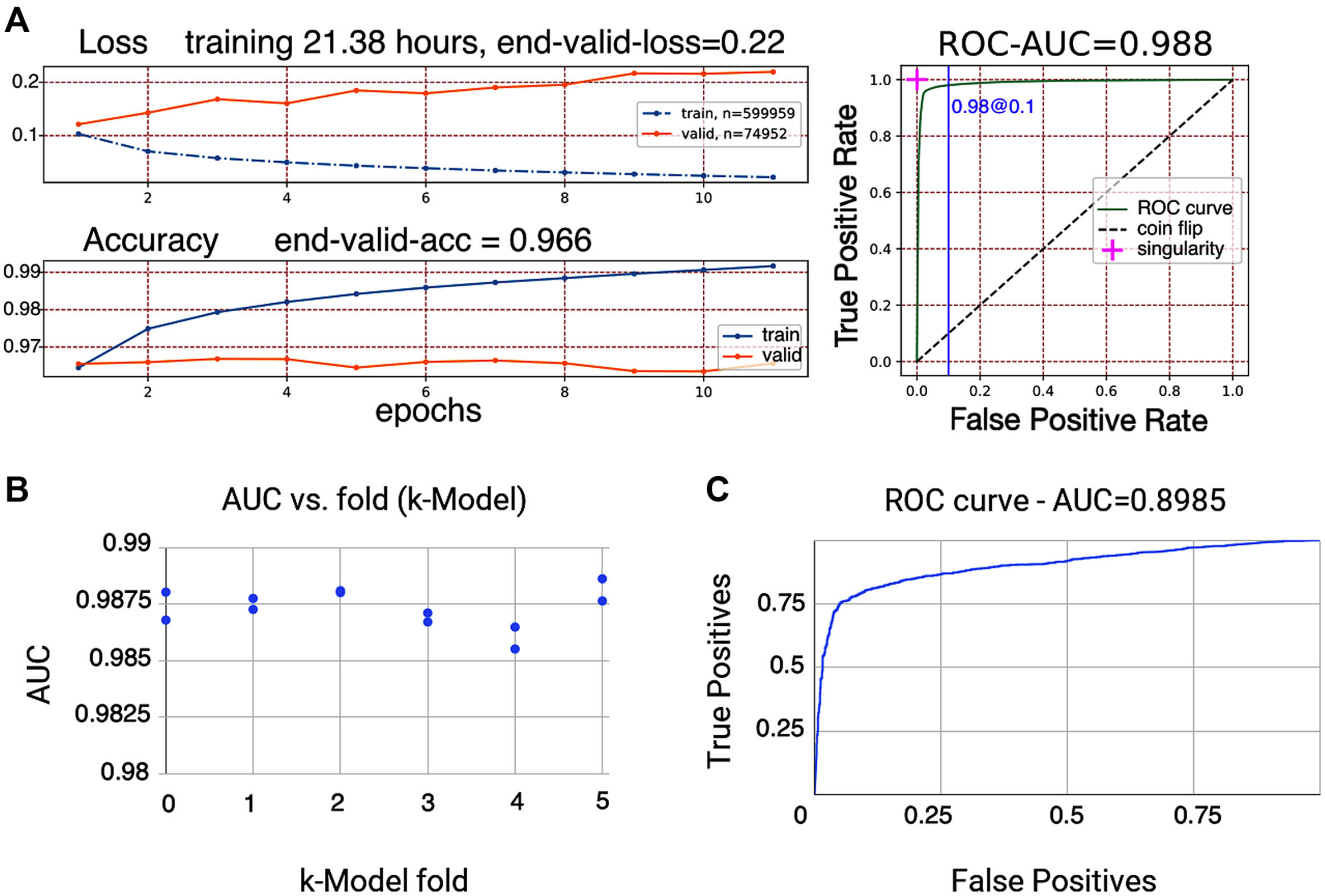
(**A**) Training convergence through the epochs. Loss and accuracy are shown as a function of epochs. (**B**) Training was performed 12 times on the plasmid-chromosome (ACLAME + PLSDB + refseq) dataset to derive 12 *k*-fold models (two per validation segment). All *k*-Fold models achieved an accuracy (AUC) on the validation segment of over 0.98 with a small statistical variance in the prediction accuracy. (**C**) The ROC-AUC curve (TPs versus FPs) on the IMG test dataset with 3280 scaffolds of length 1k–330k bases is 0.8985.

We used the *k*-fold cross-validation method, setting *k* = 6, with five data segments merged as the ‘training’ set and one validation segment that provided the loss (model error) as feedback during training. The ‘test’ data set was hidden during the training. We performed 6 independent trainings, cycling the segments to allow each of six segments to influence a different model θ_*k*_. Figure 1 illustrates the *k*-fold training method. Each model was trained for 30 epochs, until it converged, as shown in Figure 2A.

### Model topology

We have used the LSTM-based network (46) to transform the one-hot encoded nucleotide sequence into a one-dimensional vector. The left branch is made out of two LSTMs, accepts a 300 bp nucleotide sequence *x*_seq_, and compresses information into a vector of 40 features. The right branch is fully connected, accepts the feature vector *x*_f_, and produces a vector of 100 features. Both outputs are concatenated and passed to another block of fully connected layers whose output is one value—the Deeplasmid score *y* (Eq. 1). This model was implemented in Keras (47) with Tensorflow 1.3.0 (48) as the backend. The deep learning model architecture is shown in Supplementary Figures S1–S4.

### Prediction for one 300 bp sequence

For each *k*-fold we saved two models, resulting in 12 different saved models. This was done to reduce the effects of random variance in the predictions, as well as to ensure that the results were reproducible for each *k*-fold. To make a prediction on a 300 bp sequence we ran the sequence through all 12 models and then averaged the score:

(2) $$\begin{equation*}\hat y = \mathop \sum \limits_{i = 1}^{12} \;\;y\left( {\theta i} \right)\;/\;12.\end{equation*}$$

The results are shown in Supplementary Table S3.

### Prediction for one scaffold

One scaffold is sampled 50–100 times and for each 300 bp sequence the average score is computed as in (Eq. 2). Next, the scaffold-average score (*y*_avr_) and its standard deviation (σ) are computed. We allow for three-way classification as ‘plasmid’, ‘chromosome’ or ‘ambiguous’:

- if *y*_avr_ > 0.5 + 2σ then plasmid
- else if *y*_avr_ < 0.5 – 2σ then chromosome
- else ambiguous

### DNA extraction and Oxford Nanopore sequencing

We validated our plasmid computational prediction using genome re-sequencing with Oxford Nanopore long read sequencing. We used strain *Yersinia ruckeri* ATCC 29473 (a gift from Dr. Yasuo Yoshikuni). Bacteria grew in the final volume of 2 l Luria Broth until OD_600_ 0.3 was reached. DNA was extracted with Qiagen Genomic Tip 100/G (Cat No./ID: 10243) and Genomic DNA Buffer Set (Qiagen, Cat No./ID: 19060), by following suggested instructions. DNA concentration and quality were tested with Nanopore, Qubit and TapeStation. Prior sequencing samples were prepared with Ligation Sequencing Kit (SQK-LSK109) and Native Barcoding Expansion 1–12 (EXP-NBD104) and finally sequenced by Oxford Nanopore MinION. Reads were assembled using Canu 2.0 (49). Reads were also assembled in parallel by Shasta 0.6.0 (50). The 3 754 417 bp circular DNA and the 102 560 circular DNA were found from the Canu and Shasta assemblies, respectively. To map linear scaffolds of *Yersinia ruckeri* ATCC 29473 to the newly assembled plasmids, BLASTN was used, with a bitscore cutoff of 30 000. The BLASTN hits were visualized using DNAFeaturesViewer (51). The IMG scaffold names were shortened in Figure 4; all scaffolds displayed are prefixed by ‘Ga0059170_’, e.g. scaffold 114 is named ‘Ga0059170_114’ in the IMG database. Scaffold ‘Ga0059170_103’ coordinates 1–145 000 were mapped to the newly found plasmid (fragment is at 8 o’clock in Figure 4, marked 103*), away from the rest of scaffold 103 (12 to 5 o’clock in Figure 4). This subsequence is small enough to be processed by Deeplasmid and was thereby re-ran through Deeplasmid to be predicted as a separate piece of DNA. Annotation (Figure 4C) was performed based on IMG scaffold annotations (i.e. IMG annotation of scaffolds Ga0059170_112 and Ga0059170_113). Mapping of fastq reads to assembled plasmid (Supplementary Figure S6) was performed using Minimap2 (52) with SAM format output.

## RESULTS

### Training and feature selection

We constructed a training dataset of 43 094 chromosomes from 930 bacterial and 45 archaeal genera that was retrieved from Refseq, and we retrieved 27 487 plasmids from ACLAME and PLSDB (Materials and Methods). Features included sequence-related physical features and features related to the genetic content of the contig (Table 1, Supplementary Tables S1 and S2, Materials and Methods). The first feature group includes gc_content, repeats: number and size of the longest homopolymers, and total length of the sequence. The second feature group includes boolean variables of whether the sampled sequence shares similarity with chromosomal genes, chromosomal protein domains, plasmidic genes, plasmidic protein domains or origin of replication sequences (Supplementary Tables S1 and S2 and Supplementary Figure S5). For example, ribosomal proteins are usually carried on chromosomes and plasmid replication and mobilization genes are carried on plasmids. This second feature group of features includes the presence or absence of plasmid-enriched or chromosomal Pfam domains. We also included in this group the number of genes carried on the scaffold, the percent of the scaffold which is coding, and the size of the genes (‘polypeptide_aa_avg_len’) as through manual inspection we noted that plasmids tend to have more intergenic regions and smaller genes than chromosomes.

### Deeplasmid cross-validation

The training dataset was divided into six segments (Materials and Methods). One segment, called ‘validation’, was used for validating a model's training. Twelve models were trained in total, two for each selection of a validation segment. The model training over 30 epochs is characterized by an increase in prediction accuracy (defined as the ratio of correct classifications to all queries) and decrease in error (loss) on the validation segment. The Receiver Operating Characteristic (ROC) curve is shown in Figure 2A. The area under the curve (AUC) for Deeplasmid reached 0.988. To be consistent with the standard definition of AUC we have forced predictions to be binary by reducing the standard deviation σ to 0 and setting threshold θ to 0.5. The predictions made by Deeplasmid are averaged over 12 models (Materials and Methods). Figure 2B shows prediction accuracy individually for each of the 12 models over the ‘validation’ data segment. There is a high agreement between models.

### Feature significance

We also evaluated the significance of various sets of features by calculating the decrease in average AUC of the 12 models on the training data set after setting the features to zero values (thus knocking them out). We retrained the model on various combinations of features. Most of the runs where features were removed resulted in a mean AUC drop of >3 standard deviations away from the mean AUC achieved when using all features. The use of all features resulted in a mean AUC over 12 training segments of 0.987 with a relatively small standard deviation (over 12 runs) of 0.0008. The mean AUC dropped to 0.897 when not using the Pfams. The mean AUC dropped further by 5% to 0.847 after removing the hits to plasmid and chromosome-specific genes demonstrating the significance of this feature in classification of annotated contigs. When using only hits to plasmid and chromosome-specific genes (in addition to the sequence data) the mean AUC also dropped to 0.8548. Other features, each one separately, such as sequence length, homopolymer-related features (the longest homopolymer and the total number of homopolymers of length > 5), and gene density in the scaffold had relatively little contribution. However, we trained the model with all features since removing features translated to an increase in the error of prediction. We provide an analysis of the mean AUCs observed over 12 models with various feature sets (Supplementary Table S4). We conclude that using curated biological information provides a clear advantage over previous algorithms, which only used ‘physical’ features of the sequences (such as gc content and scaffold length).

### Testing Deeplasmid on independent dataset: isolate genomes from IMG database

To test Deeplasmid, we used an independent dataset retrieved from IMG database (53,54). We downloaded the sequences of 1834 isolate genomes that have at least one replicon annotated as plasmid. This set included a total of 6820 scaffolds and contigs, with 3093 of them annotated as plasmids and 3727 annotated as chromosomes. Similar to our training methodology, the IMG test dataset was reduced to 4758 scaffolds of length 1k–330k bases, while scaffolds outside this length range were classified as either too long or too short. For testing on independent data, we removed from the IMG test dataset any sequence that had 90% identity along 90% of length coverage with any sequence in the training dataset, leaving us with 3280 sequences: 1500 chromosomal and 1780 plasmids. Figure 2C shows the ROC curve for the IMG test dataset, achieving an AUC of 0.8985 on the independent dataset. This indicates that the trained Deeplasmid model is applicable widely, and does not suffer from overfitting. We noted that several features could differentiate well between plasmids and chromosomes in the test set, including “genes per Mb”, hits to plasmid genes, hits to plasmid Oris, and plasmid Pfam hits count (Supplementary Figure S5).

**Figure 3.**
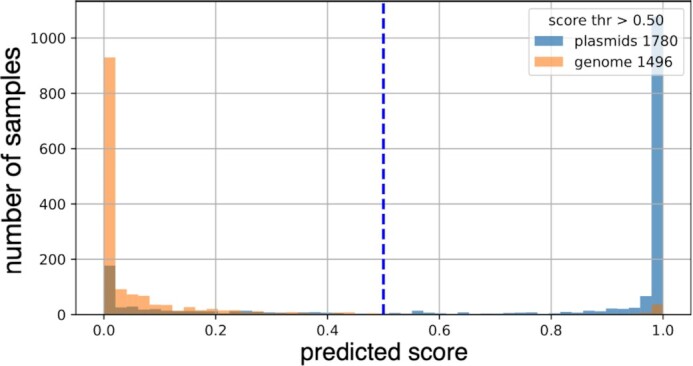
Evaluation on the IMG test dataset. The class separation is clear based on a threshold of 0.5. The percent of classifications above the threshold that are plasmids is 94.45% (precision). The percent of all plasmids classified above the threshold is 75.56% (recall). The percent of all chromosomes that are classified below the threshold (specificity) is 94.46%.

Figure 3 shows the counts of plasmid and chromosomal scaffolds assigned a certain score by Deeplasmid. Setting the threshold for separating the two classes at 0.5, the precision or purity of the predicted positive class (plasmid sequences) is 94.45%. On the other hand, recall is 75.56% (details in Supplementary Info) indicating that the DL model missed some plasmids, classifying them as chromosomal fragments. Deeplasmid did not classify four chromosomal sequences since they contained N bases.

We used this dataset to compare the performance of our Deeplasmid model to the comparable state-of-the-art tools PlasFlow (17), cBar (16), mlplasmids (22), Platon (23), and PlasmidVerify (24) (Table 2). Deeplasmid achieved a precision of 94.45% on the test dataset compared to precisions of 94.16% for Platon, 92% for PlasmidVerify, 80.9% for PlasFlow, 77.5% for cBar, and 55–70% for mlplasmids (Table 3). The overall accuracy, measuring the correct predictions over the entire dataset is 84.2% for Deeplasmid, which is higher than PlasmidVerify (82%), Platon (78%), cBar (75%), PlasFlow (62%) or mlplasmids (52–64%). Deeplasmid's specificity is 94.46%, which is a comparable success rate for chromosome identification to those of PlasmidVerify (93%) and Platon (95.33%). Deeplasmid did not classify four chromosomes with ‘*N*’ bases, significantly less than PlasFlow's 394 unclassified chromosomes that affected PlasFlow's specificity and accuracy metrics. This comparison demonstrates that addition of biological features vastly improves the performance of programs with otherwise similar inputs and goals.

**Table 2.** Comparison of Deeplasmid to PlasmidVerify, PlasFlow, cBar, Platon and mlplasmids on the IMG test dataset. For mlplasmids we tested both the Enterococcus model, since it was the most populous genus in the test data, and *E. coli* model. For Deeplasmid we removed from the IMG test dataset any sequence that had 90% identity at 90% sequence length coverage with any sequence of the ACLAME + refseq.bacteria + PLSDB training dataset

	Plasmids (1780)				Chromosomes (1500)			
Truth: Classified as:	Plas. (TP)	Chrom. (FN)	Ambig. (FN)	Total FN	Chrom. (TN)	Plas. (FP)	Ambig.	Total contigs
Deeplasmid	1345	435	0	435	1417	79	4	3280
PlasmidVerify	1294	486	0	486	1398	102	0	3280
PlasFlow	1219	161	400	561	819	287	394	3280
cBar	1395	385	0	385	1096	404	0	3280
Platon	1130	650	0	650	1430	70	0	3280
mlplasmids /Ent	1055	725	0	725	1061	439	0	3280
mlplasmids /Eco	1037	743	0	743	668	832	0	3280

**Table 3.** Performance of the difference plasmid prediction algorithms. For plasmids we used the *Enterococcus faecium* (Ent) and *E. coli* (Eco) models on all plasmids. In bold are the best performing tools in each category

	Precision TP/(TP + FP)	Recall(Sens.) TP/(TP + FN)	Specificity TN/(#chromosomes)	Accuracy (TP + TN)/(#contigs)
Deeplasmid	**0.9445**	0.7556	0.9446	**0.842**
PlasmidVerify	0.92	0.72	0.93	0.82
PlasFlow	0.809	0.684	0.546	0.62
cBar	0.775	**0.78**	0.73	0.75
Platon	0.9416	0.634	**0.9533**	0.78
mlplasmids /Ent	0.70	0.59	0.7	0.645
mlplasmids /Eco	0.554	0.58	0.45	0.52

The runtimes were taken on a Cray XC40 supercomputer (5 Intel Xeon ‘Haswell’ nodes with 120GB, 16 cores). The training runtime was ∼23 h for the ACLAME + PLSDB and RefSeq.bacteria dataset with 43 094 sequences. There was early stopping at 11 epochs, which translates to ∼2 h per epoch. The prediction runtime was <2 s per scaffold or under 2 min for a microbial genome assembly, assuming a typical microbial genome assembly contains 1–60 scaffolds. This satisfies the scalability requirement for an automated plasmid finding tool with Deeplasmid being potentially applicable to large-scale genomic and metagenomic data.

### Plasmid prediction reproducibility

To test how reproducible Deeplasmid predictions are, we compared its plasmid predictions from independent sequencing of highly similar strains of the same organism. We selected *Burkholderia pseudomallei* NCTC 13392, the causative agent of melioidosis. We found 10 versions of *B. pseudomallei* NCTC 13392 strain in IMG (55). These are the wildtype strain and related isolates that were isolated after 1–4 days from mouse infection (55–57). The related genomes had similar sizes of 6.96–7.16 Mb and were assembled in 48–248 contigs (Table 4) with 5899–6016 genes. It is yet unknown whether these strains encode one or more plasmids. Deeplasmid predicted at least one plasmid with high score >0.9 in most cases (‘plasmid #1’ of at least 28 kb) for all ten genomes showing high reproducibility between genomes that were assembled differently and were only partially overlapping. It seems like a replicable plasmid with low coding density, several short genes, and genes mapped to integrase, transposes, two toxin-antitoxin systems, and several DNA manipulation genes (primase/helicase, restriction enzyme, invertase, nuclease). However, important plasmid replication genes are lacking on the scaffold, suggesting that the current sequencing and assembly status did not capture the entire plasmid in a single scaffold. Two other plasmids were predicted in only a subset of the genomes. Four genome variants had ‘plasmid #2’ (6.2–7.4 kb) predictions that include a transposase gene. The wildtype strain, genome ID 2551306633, had ‘plasmid #3’ (18.7 kb), with the maximal Deeplasmid score (1.00) and a RepA plasmid replication plasmid. It is yet unknown whether predicted plasmids #2 and #3 are bona fide and their presence varies between related strains that are exposed to selection during host infection, whether the sequencing and assembly did not capture these in all cases (leading to polymorphism), or whether these are false positive predictions. Overall, we see strong replicability in Deeplasmid predictions.

**Table 4.** Plasmid prediction in ten genomes that were derived from *Burkholderia pseudomallei* NCTC 13392

IMG genome ID	# contigs	Predicted plasmid #1 Marker genes: integrase, transposase, toxin-antitoxins. scaffold, size, score	Predicted plasmid #2 Marker gene: transposase. scaffold, size, score	Predicted plasmid #3 Marker gene: RepA. scaffold, size, score
2551306687	176	D437DRAFT_AUVI01000171_1.171, 15604, 0.945	NA	NA
2551306690	158	D445DRAFT_AWEO01000008_1.8, 15076, 0.948	NA	NA
2551306691	248	D438DRAFT_AUVJ01000245_1.245, 16121, 0.95	NA	NA
2551306694	148	D439DRAFT_AUVK01000143_1.143, 16122, 0.949	NA	NA
2551306692	153	D440DRAFT_AUVL01000036_1.36, 16743, 0.857	D440DRAFT_AUVL01000132_1.132, 7408, 0.985	NA
2551306689	157	D441DRAFT_AUVM01000076_1.76, 21079, 0.981	D441DRAFT_AUVM01000141_1.141, 6269, 0.686	NA
2554235004	155	D442DRAFT_AUVN01000143_1.143, 13744, 0.948	NA	NA
2551306693	150	D443DRAFT_AUVO01000133_1.133, 13024, 0.831	D443DRAFT_AUVO01000128_1.128, 6259, 0.732	NA
2551306697	140	D444DRAFT_AUVP01000078_1.78, 18565, 0.969	D444DRAFT_AUVP01000112_1.112, 7409, 0.985	NA
2551306633	48	F520DRAFT_AOUG01000024_1.24, 28064, 0.708	NA	F520DRAFT_AOUG01000026_1.26, 18669, 1.00

### Experimental validation of a new plasmid based on Deeplasmid prediction

**Figure 4.**
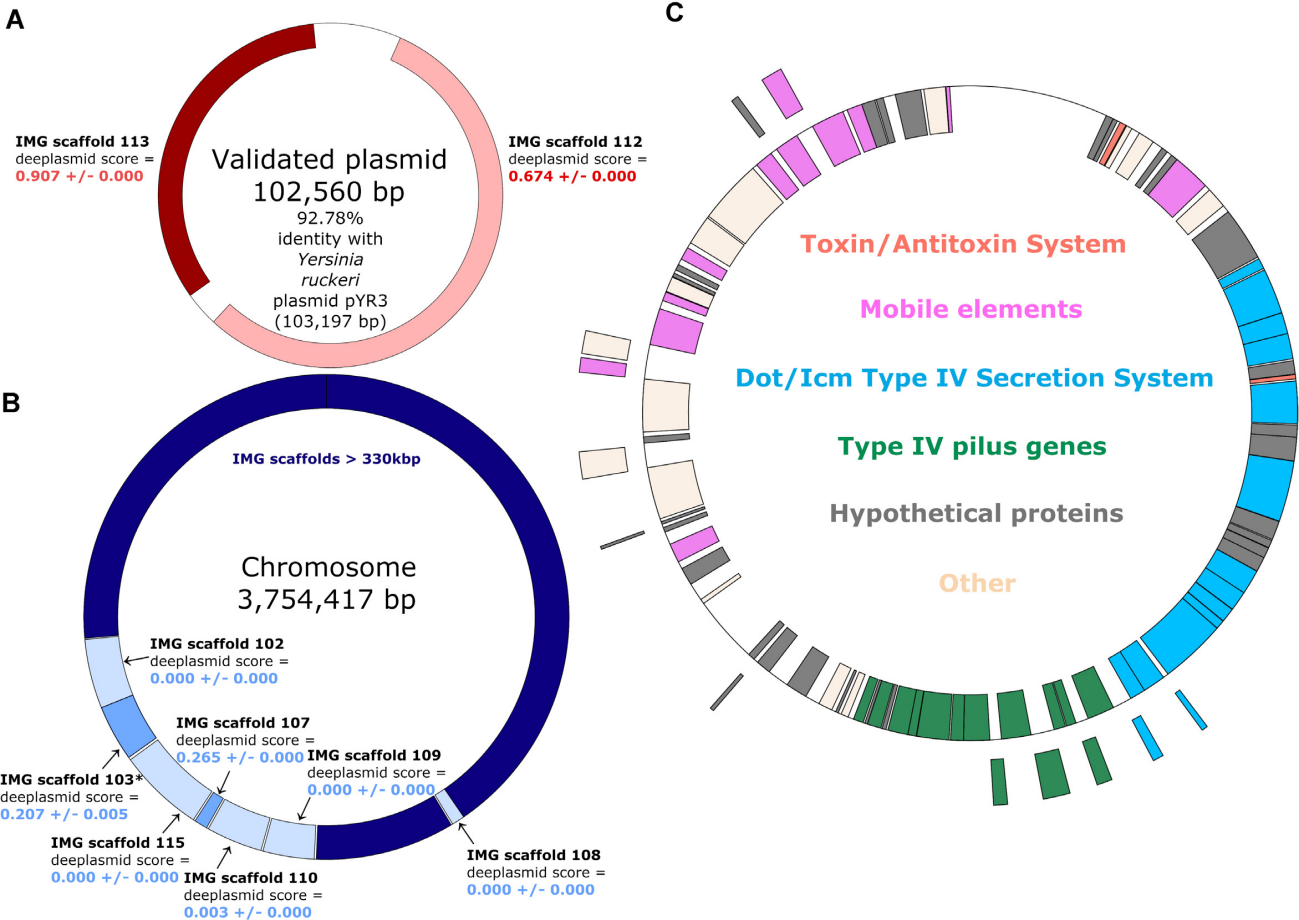
Deeplasmid validation. *Yersinia ruckeri* ATCC 29473 was sequenced with Oxford Nanopore MinION and assembled with Canu and with Shasta. (**A**) The assembled contigs included a circular piece of DNA that shares 92.78% identity with a known *Yersinia* plasmid (pYR3; Genbank: LN681230.1). Two linear scaffolds of this genome were predicted by Deeplasmid to be from plasmids (shades of red), and indeed they align with the newly-found plasmid. (**B**) The assembled contigs also contained a chromosome. Most of the linear scaffolds for this genome did not undergo Deeplasmid prediction, due to their large size (>330kb; dark navy blue). However, those within the size range were largely predicted to be chromosomal in origin (lighter shades of blue). Scaffold 103* is a subsequence of a larger IMG scaffold that matched twice to the assembled chromosome; this short region was predicted by Deeplasmid here (see Materials and Methods). (**C**) Validated plasmid gene functions. Annotations show genes that are classically associated with plasmids. Color scheme is indicated in the center; grey = ‘hypothetical protein’; tan = other functions.

In order to demonstrate Deeplasmid's ability to predict plasmids in biological samples, we focused on the fish pathogen *Yersinia ruckeri* ATCC 29473 (IMG genome ID: 2609460118). Until this work, this strain had only been sequenced by 454 and Illumina short read sequencers, resulting in 15 linear scaffolds and therefore presumably lacking a plasmid (58). Using comparative genomics, researchers studying a similar bacterial strain inferred that *Y. ruckeri* ATCC 29473 may encode a plasmid, but no long read sequencing was performed to confirm this hypothesis (59). The 15 linear scaffolds of *Y. ruckeri* ATCC 29473 were then used as input to Deeplasmid, and labeled as either plasmid or chromosomal. One long scaffold of 35 kb (IMG scaffold 113, Figure 4A) got a Deeplasmid score of 0.907, strongly suggesting that it is derived from a plasmid. In contrast, other scaffolds were either very long (e.g. IMG scaffold 103 of 1.6 Mb) or received Deeplasmid score below 0.265, suggesting that they derive from a bacterial chromosome. To validate these labels, we grew *Y. ruckeri* bacteria in the lab, extracted DNA, and sequenced it with a long read sequencing method (Oxford Nanopore Technology). In contrast to the previous short read methods, we were able to find large circular pieces of DNA. We found a ∼3.7 Mb chromosome, and a ∼102 kb plasmid (Supplementary Figure S6). Beyond the fact that it is circular, we are confident the latter piece of DNA is a plasmid since it has very high similarity to a known *Yersinia* plasmid (pYR3; Genbank: LN681230.1).

Upon mapping the linear assembled scaffolds onto the newly sequenced circular DNA fragments, we indeed find that predicted plasmid fragments map to the 102 kb plasmid, demonstrating the predictive power of Deeplasmid and its ability to detect large plasmids in genomic data (Figure 4A). We note that a number of the linear scaffolds for this genome did not undergo Deeplasmid prediction due to their large size (>330 kb). Exactly because of their large size, they are assumed to be chromosomal in origin. However, those within the size range of Deeplasmid functionality (1–330 kb) were largely predicted to be chromosomal in origin (Figure 4B).

Looking at the functions of the genes carried on the plasmid, we see many genes previously found on similar plasmids (59), and that have plasmid-related functions. We identified Type IV pilus genes, which may be used for transfer of plasmid from one cell to another (60), or possibly used as a virulence factor (59). Also encoded on the plasmid is the Type IV Secretion System, which may also be involved in plasmid transfer (61) and/or virulence (59). We also detected on the plasmid RelE, a toxin which is commonly found in plasmid addiction systems (62). Furthermore, we found mobilization genes like transposon genes, integrases, and DNA recombinases. Overall, we conclude that this is a *bona fide* plasmid based on its circularity, separation from the main chromosome, similarity to known plasmids, and plasmidic gene content.

## DISCUSSION

In this work, we provide an accurate algorithm to classify assembled contigs or scaffolds generated by any sequencing platform and assembly algorithm, as parts of plasmids and chromosomes using a deep learning approach. Importantly, Deeplasmid was the most accurate when compared to five other plasmid prediction tools. By training deep learning models on the specific features of plasmids and chromosomes, we have shown that it is possible to efficiently separate plasmids from chromosomal sequences. While physical sequence features can be used to predict if a sequence is of plasmid or chromosome origin, the DNA sequence itself improves the deep learning models by keeping a memory of what came earlier in the sequence. The reason is that statistical features of sequence composition, such as GC content or oligonucleotide profiles, fail to capture the nucleotide composition over the length of the sequence, as explained in earlier work (8). One of the reasons why our Deeplasmid model has better precision at predicting plasmids than other methods is that it averages predictions over multiple 300 bp windows sampled over the length of the sequence, instead of analyzing contigs and scaffolds as a single DNA molecule. As a result, each prediction for a 300 bp sequence contributes to the overall result. Additionally, we complemented sequences with extracted feature data that enhance the prediction accuracy. The plasmid-specific ORIs, Pfams, and genes, as well as chromosome-specific Pfams and genes serve as essential data features. Averaging the predictions over many 300-base sequences and including biologically meaningful features resulted in a better ability to classify a sequence as a plasmid or chromosome.

Just like other methods that rely on the nucleotide composition signatures of plasmids and chromosomes, whether as hidden or extracted features, Deeplasmid is likely to have problems with very short sequences for which it may have trouble obtaining a proper sequence signature. For this reason, we limited the sequences used in the model construction to those of minimum 1 kb length. There are also very few existing plasmids that are larger than 330 kb and therefore we could not train our algorithm on these megaplasmids.

Prediction of plasmids is complicated in large genomic datasets with possible chromosomal integrations of plasmids. Current challenges in the field of plasmid identification include discriminating chromosomes that resemble plasmid sequences or plasmids with chromosomal replication genes. Another challenge is to achieve high prediction accuracy on unknown or understudied microbial lineages that may contain exotic plasmids, since any machine learning tool will be trained with the current knowledge. The chromosomes and plasmids used in the training step are biased towards the most studied lineages such as proteobacteria, actinobacteria, firmicutes, and bacteroidetes. However, we hypothesize that using the large set of Pfam domains will allow classification of plasmidic or chromosomal scaffolds that exist beyond these phyla.

Deeplasmid predicts plasmids with a low false positive error rate using only an assembled fasta file as input. It can identify both circular and linear plasmids. The output is a per-scaffold classification of chromosomal, plasmid, or ambiguous contig, along with a score representing the confidence of the prediction. Although the default probability threshold for separation of classes was set at 0.5 based on our benchmarking, users can specify their own filtering cutoffs. Deeplasmid out-performed other available tools in terms of accuracy for single microbial assembly plasmidome analyses. Moreover, we provided experimental evidence for a new plasmid that was predicted using Deeplasmid. A future research direction is to employ Deeplasmid for identification of plasmids in large-scale metagenomic data from different environments or to uncover novel plasmid-borne antimicrobial resistance genes or novel microbial genes that are horizontally transferred via plasmids. This and myriad other high impact applications of Deeplasmid are possible due to its fast running time and scalability.

## SUPPLEMENTARY INFORMATION

The Supplementary Information file includes Deeplasmid running instructions, comments on features used in chromosome/plasmid classification, Code Repository, Software Design, Deep learning model architecture, AUC per fold on the training dataset, ROC curve for the IMG test dataset, and other data.

## DATA AVAILABILITY

The software is available with a BSD license: https://github.com/wandreopoulos/deeplasmid. A Docker container is available on DockerHub under: https://hub.docker.com/r/billandreo/deeplasmid. The raw reads from the Oxford Nanopore sequencing project that was used to validate a new plasmid in *Yersinia ruckeri* were deposited to NCBI’s Sequence Read Archive (SRA) under study PRJNA721367.

## Supplementary Material

gkab1115_Supplemental_Files

## Notes

Present address: Miriam Lucke, Department of Molecular Biology, Max Planck Institute for Developmental Biology, Spemannstr. 35, 72076 Tübingen, Germany.
