# Supplementary material for: Deeplasmid: deep learning accurately separates plasmids from bacterial chromosomes
Source: Nucleic Acids Res. 2021 Dec 6;50(3):e17. doi: 10.1093/nar/gkab1115 (PMC8860608; doi:10.1093/nar/gkab1115)
Supplement: gkab1115_Supplemental_Files [file gkab1115_supplemental_files.zip › Deeplasmid - Supplementary Information - proof.docx]

Deeplasmid - Supplementary Information

For questions contact:

Bill Andreopoulos, [wandreopoulos@lbl.gov](mailto:wandreopoulos@lbl.gov)

Asaf Levy alevy@mail.huji.ac.il

Table of Contents:

[Instructions to run on a Mac or Linux-based computer:](#_1f2syi30o146)

[Example commands for run on Mac with Docker:](#_7zl6pyc4jlsg)

[Building the Docker image](#_i5ajad8yqw2d)

[First issue - include Prodigal in the built container image:](#_3738q0pkcgw8)

[Second issue - bbmap needs to be in the built Docker image.](#_84bwnda6gjes)

[Third issue - the plasmid trained models need to be placed in the Docker image since they are not in the code repository](#_i375og6qdia1)

[Supplementary Tables](#_sovpk9c65acx)

[AUC per k-fold and average for combinations of features](#_wb0lcs35w3z1)

[Code Repository](#_valoyjfiiwzp)

[Software Design](#_hlnrezi8f6rf)

[The training and test datasets](#_8uzgkb8pyi37)

[Supplementary Figures](#_s512l8bhr853)

[Deep learning model architecture](#_yj3yz8n3vix2)

[Difference of features between chromosomal DNA and plasmids](#_h3cmxre2155)

[Yersinia assembly of circular plasmid](#_yvr0exf1oe8m)

[References](#_k9e0erndecij)

### Instructions to run on a Mac or Linux-based computer:

The deeplasmid software runs on Mac with docker. After installing docker, please run:

docker login

docker pull billandreo/deeplasmid

docker run -it -v /path/to/input/fasta:/srv/jgi-ml/classifier/dl/in.fasta -v /path/to/output/directory:/srv/jgi-ml/classifier/dl/outdir billandreo/deeplasmid feature_DL_plasmid_predict.sh in.fasta outdir

Change the blue highlighted paths above to point to the absolute paths for your input fasta and the output directories. An example run:

docker run -it -v /Users/X/Downloads/jgi-ml/classifier/dl/temp/649989979.fna:/srv/jgi-ml/classifier/dl/in.fasta -v /Users/X/Downloads/jgi-ml/classifier/dl/temp/649989979.fna.OUT:/srv/jgi-ml/classifier/dl/outdir billandreo/deeplasmid feature_DL_plasmid_predict.sh in.fasta outdir

#### Example commands for run on Mac with Docker:

The two parameters that a user needs to set when running with Docker are the path to input fasta and path to output directory:

Williams-MBP:dl andreopo$ pwd

/Users/andreopo/Downloads/jgi-ml_DOCKER_CLEANEDUP/classifier/dl

Williams-MBP:dl andreopo$ docker run -it -v /Users/andreopo/Downloads/jgi-ml_COMMITTED/classifier/dl/temp/649989979.fna:/srv/jgi-ml/classifier/dl/in.fasta -v /Users/andreopo/Downloads/jgi-ml_DOCKER_CLEANEDUP/classifier/dl/temp/649989979.fna.OUT:/srv/jgi-ml/classifier/dl/outdir billandreo/deeplasmid feature_DL_plasmid_predict.sh in.fasta outdir

A user can check the predictions in the predictions.txt output file:

Williams-MBP:dl andreopo$ cat temp/649989979.fna.OUT/outPR.20201008_211704/predictions.txt

name,pred,conf

NZ_ADHJ01000001,LONGER_330000.0,1

NZ_ADHJ01000014,LONGER_330000.0,1

NZ_ADHJ01000017,LONGER_330000.0,1

NZ_ADHJ01000025,LONGER_330000.0,1

NZ_ADHJ01000027,SHORTER_1000.0,1

NZ_ADHJ01000030,SHORTER_1000.0,1

NZ_ADHJ01000032,SHORTER_1000.0,1

NZ_ADHJ01000037,LONGER_330000.0,1

NZ_ADHJ01000051,SHORTER_1000.0,1

nz_adhj01000006 paenibacillus vortex v453 cnt_pvor1000006, whole genome shotgun sequence.,GENOME,0.019 +/- 0.001

nz_adhj01000002 paenibacillus vortex v453 cnt_pvor1000002, whole genome shotgun sequence.,GENOME,0.171 +/- 0.004

nz_adhj01000034 paenibacillus vortex v453 cnt_pvor1000034, whole genome shotgun sequence.,GENOME,0.134 +/- 0.005

nz_adhj01000013 paenibacillus vortex v453 cnt_pvor1000013, whole genome shotgun sequence.,GENOME,0.011 +/- 0.001

nz_adhj01000041 paenibacillus vortex v453 cnt_pvor1000041, whole genome shotgun sequence.,PLASMID,0.921 +/- 0.001

nz_adhj01000007 paenibacillus vortex v453 cnt_pvor1000007, whole genome shotgun sequence.,GENOME,0.005 +/- 0.001

...

...

### Building the Docker image

To build the Docker image, please use:

docker build -t billandreo/deeplasmid -f Dockerfile.v2 .

There are a few issues that need to be addressed when building the Docker image:

#### First issue - include Prodigal in the built container image:

git clone <https://github.com/hyattpd/Prodigal.git>

With this command you can enter into the Docker image:

docker run -it billandreo/deeplasmid /bin/bash

Test if prodigal runs with:

cd Prodigal/

./Prodigal/prodigal

#### Second issue - bbmap needs to be in the built Docker image.

Please check bbmap out from sourceforge into the local dl directory. Commands to place bbmap in the docker image - first place bbmap tar.gz file under the local dl dir:

mv ../../../BBMap_38.73.tar.gz .

gunzip BBMap_38.73.tar.gz

tar xvf BBMap_38.73.tar

When you build the Docker image with Dockerfile.v2, as shown above, BBMap files will then be placed in the deeplasmid image with the CP command that’s in the Dockerfile.v2

With this command you can enter into the Docker image:

docker run -it billandreo/deeplasmid /bin/bash

This is bbmap version in the Docker container:

-rw-r--r-- 1 root root 34440192 Jan 1 2020 BBMap_38.73.tar

drwxr-xr-x 9 root root 12288 Jan 4 2020 bbmap

#### Third issue - the plasmid trained models need to be placed in the Docker image since they are not in the code repository

For space reasons the models are not in the code repo. Under the local dl directory:

cd Plasmid_Models/

scp -r /data/Plasmid_Models/plasmid4z-newfeat5* .

Those will then be placed in the deeplasmid image with the CP command that’s in the Dockerfile.v2

Note: for the model you only need .h5 files under Plasmid_Models, which are of a reasonable size. You don’t need the data subdirectories, which are in the GB size range and can cause the Docker container size to become very large. The Plasmid_Models.tar.gz (5MB) can also be sent as email attachment.

###

### **Supplementary Tables**

**Supplementary Table S1. List of features used, and their sources, excluding the Pfam domains**

| **Features** | **Comment** | **Reference** |
| --- | --- | --- |
| Total length of the sequence | Chromosomes tend to be larger than plasmids. |  |
| %GC of the sequence |  | [(1)](https://paperpile.com/c/n3gJtb/cZyT) |
| Longest A homopolymer |  |  |
| Longest C homopolymer |  |  |
| Longest G homopolymer |  |  |
| Longest T homopolymer |  |  |
| Count of A homopolymer longer than 5 bp |  |  |
| Count of C homopolymer longer than 5 bp |  |  |
| Count of G homopolymer longer than 5 bp |  |  |
| Count of T homopolymer longer than 5 bp |  |  |
| Contig coding frequency (coding bases/size) | Based on manual inspection of several plasmids, they seem to have lower coding frequency than chromosomes. |  |
| Count of oriV | Plasmid origin of replication | [(2)](https://paperpile.com/c/n3gJtb/3f9r4) |
| Count of origin of replication of different plasmids from gram+ and gram- bacteria | Matches to genbank AB015179.1, DQ459477.1, AF239690.1, AF239689.1, X12587.1, EF546766.1, DQ439974.1, V00270.1, Y08803.1, Y08791.1, Y08790.1, X70131.1, V01374.1, V00327.1 |  |
| Count of TrfA gene | Plasmid gene | [(3)](https://paperpile.com/c/n3gJtb/Xw1hw) |
| Count of DNA replication initiator gene (DnaA) | Chromosomal gene | [(4)](https://paperpile.com/c/n3gJtb/B9oBE) |
| Count of oriC | Chromosomal origin of replication |  |
| Count of Rep gene | Plasmid gene | [(5)](https://paperpile.com/c/n3gJtb/0aoMX) |
| Count of COG0018 | Chromosomal essential gene | [(6, 7)](https://paperpile.com/c/n3gJtb/VE4Tm+1Pjom) |
| Count of COG0008 | Chromosomal essential gene | [(6–8)](https://paperpile.com/c/n3gJtb/VE4Tm+1Pjom+LU0Cl) |
| Count of COG0124 | Chromosomal essential gene | [(6, 7)](https://paperpile.com/c/n3gJtb/VE4Tm+1Pjom) |
| Count of COG0495 | Chromosomal essential gene | [(6, 7)](https://paperpile.com/c/n3gJtb/VE4Tm+1Pjom) |
| Count of COG0442 | Chromosomal essential gene | [(6, 7)](https://paperpile.com/c/n3gJtb/VE4Tm+1Pjom) |
| Count of COG0172 | Chromosomal essential gene | [(6, 7)](https://paperpile.com/c/n3gJtb/VE4Tm+1Pjom) |
| Count of COG0090 | Chromosomal essential and unclonable gene | [(6–8)](https://paperpile.com/c/n3gJtb/VE4Tm+1Pjom+LU0Cl) |
| Count of COG0087 | Chromosomal essential and unclonable gene | [(6–8)](https://paperpile.com/c/n3gJtb/VE4Tm+1Pjom+LU0Cl) |
| Count of COG0088 | Chromosomal essential and unclonable gene | [(6–8)](https://paperpile.com/c/n3gJtb/VE4Tm+1Pjom+LU0Cl) |
| Count of COG0097 | Chromosomal essential gene | [(6, 7)](https://paperpile.com/c/n3gJtb/VE4Tm+1Pjom) |
| Count of COG0102 | Chromosomal essential gene | [(6, 7)](https://paperpile.com/c/n3gJtb/VE4Tm+1Pjom) |
| Count of COG0092 | Chromosomal essential gene | [(6, 7)](https://paperpile.com/c/n3gJtb/VE4Tm+1Pjom) |
| Count of COG0522 | Chromosomal essential gene | [(6, 7)](https://paperpile.com/c/n3gJtb/VE4Tm+1Pjom) |
| Count of COG0098 | Chromosomal essential gene | [(6, 7)](https://paperpile.com/c/n3gJtb/VE4Tm+1Pjom) |
| Count of COG0202 | Chromosomal essential gene | [(6, 7)](https://paperpile.com/c/n3gJtb/VE4Tm+1Pjom) |
| Count of COG0592 | Chromosomal essential and unclonable gene | [(6–8)](https://paperpile.com/c/n3gJtb/VE4Tm+1Pjom+LU0Cl) |
| Count of COG0037 | Chromosomal essential gene | [(6, 7)](https://paperpile.com/c/n3gJtb/VE4Tm+1Pjom) |
| Count of COG0201 | Chromosomal essential gene | [(6, 7)](https://paperpile.com/c/n3gJtb/VE4Tm+1Pjom) |
| Count of COG0552 | Chromosomal essential gene | [(6, 7)](https://paperpile.com/c/n3gJtb/VE4Tm+1Pjom) |
| Count of COG0462 | Chromosomal essential gene | [(6, 7)](https://paperpile.com/c/n3gJtb/VE4Tm+1Pjom) |
| Count of COG0593 | Chromosomal unclonable gene | [(8)](https://paperpile.com/c/n3gJtb/LU0Cl) |
| Count of COG0776 | Chromosomal unclonable gene | [(8)](https://paperpile.com/c/n3gJtb/LU0Cl) |
| Count of COG0592 | Chromosomal unclonable gene | [(8)](https://paperpile.com/c/n3gJtb/LU0Cl) |
| Count of COG0206 | Chromosomal unclonable gene | [(8)](https://paperpile.com/c/n3gJtb/LU0Cl) |
| Count of COG0234 | Chromosomal unclonable gene | [(8)](https://paperpile.com/c/n3gJtb/LU0Cl) |
| Count of COG2885 | Chromosomal unclonable gene | [(8)](https://paperpile.com/c/n3gJtb/LU0Cl) |
| Count of COG3203 | Chromosomal unclonable gene | [(8)](https://paperpile.com/c/n3gJtb/LU0Cl) |
| Count of COG0048 | Chromosomal unclonable gene | [(8)](https://paperpile.com/c/n3gJtb/LU0Cl) |
| Count of COG1278 | Chromosomal unclonable gene | [(8)](https://paperpile.com/c/n3gJtb/LU0Cl) |
| Count of COG0052 | Chromosomal unclonable gene | [(8)](https://paperpile.com/c/n3gJtb/LU0Cl) |
| Count of COG0050 | Chromosomal unclonable gene | [(8)](https://paperpile.com/c/n3gJtb/LU0Cl) |
| Count of COG0228 | Chromosomal unclonable gene | [(8)](https://paperpile.com/c/n3gJtb/LU0Cl) |
| Count of COG1028 | Chromosomal unclonable gene | [(8)](https://paperpile.com/c/n3gJtb/LU0Cl) |
| Count of COG1959 | Chromosomal unclonable gene | [(8)](https://paperpile.com/c/n3gJtb/LU0Cl) |
| Count of COG3104 | Chromosomal unclonable gene | [(8)](https://paperpile.com/c/n3gJtb/LU0Cl) |
| Count of COG0739 | Chromosomal unclonable gene | [(8)](https://paperpile.com/c/n3gJtb/LU0Cl) |
| Count of COG0594 | Chromosomal unclonable gene | [(8)](https://paperpile.com/c/n3gJtb/LU0Cl) |
| Count of COG0774 | Chromosomal unclonable gene | [(8)](https://paperpile.com/c/n3gJtb/LU0Cl) |
| Count of COG1077 | Chromosomal unclonable gene | [(8)](https://paperpile.com/c/n3gJtb/LU0Cl) |
| Count of COG0716 | Chromosomal unclonable gene | [(8)](https://paperpile.com/c/n3gJtb/LU0Cl) |
| Count of COG0799 | Chromosomal unclonable gene | [(8)](https://paperpile.com/c/n3gJtb/LU0Cl) |
| Count of COG1734 | Chromosomal unclonable gene | [(8)](https://paperpile.com/c/n3gJtb/LU0Cl) |
| Count of COG0089 | Chromosomal unclonable gene | [(8)](https://paperpile.com/c/n3gJtb/LU0Cl) |
| Count of COG0849 | Chromosomal unclonable gene | [(8)](https://paperpile.com/c/n3gJtb/LU0Cl) |
| Count of COG1686 | Chromosomal unclonable gene | [(8)](https://paperpile.com/c/n3gJtb/LU0Cl) |
| Count of COG1399 | Chromosomal unclonable gene | [(8)](https://paperpile.com/c/n3gJtb/LU0Cl) |
| Count of COG0236 | Chromosomal unclonable gene | [(8)](https://paperpile.com/c/n3gJtb/LU0Cl) |
| Count of COG1414 | Chromosomal unclonable gene | [(8)](https://paperpile.com/c/n3gJtb/LU0Cl) |
| Count of COG0332 | Chromosomal unclonable gene | [(8)](https://paperpile.com/c/n3gJtb/LU0Cl) |
| Count of COG0527 | Chromosomal unclonable gene | [(8)](https://paperpile.com/c/n3gJtb/LU0Cl) |
| Count of COG0102 | Chromosomal unclonable gene | [(8)](https://paperpile.com/c/n3gJtb/LU0Cl) |
| Count of COG0361 | Chromosomal unclonable gene | [(8)](https://paperpile.com/c/n3gJtb/LU0Cl) |
| Count of COG0568 | Chromosomal unclonable gene | [(8)](https://paperpile.com/c/n3gJtb/LU0Cl) |
| Count of COG0764 | Chromosomal unclonable gene | [(8)](https://paperpile.com/c/n3gJtb/LU0Cl) |
| Count of COG0199 | Chromosomal unclonable gene | [(8)](https://paperpile.com/c/n3gJtb/LU0Cl) |
| Count of COG0845 | Chromosomal unclonable gene | [(8)](https://paperpile.com/c/n3gJtb/LU0Cl) |
| Count of COG0545 | Chromosomal unclonable gene | [(8)](https://paperpile.com/c/n3gJtb/LU0Cl) |
| Count of COG0261 | Chromosomal unclonable gene | [(8)](https://paperpile.com/c/n3gJtb/LU0Cl) |
| Count of COG0834 | Chromosomal unclonable gene | [(8)](https://paperpile.com/c/n3gJtb/LU0Cl) |
| Count of COG0189 | Chromosomal unclonable gene | [(8)](https://paperpile.com/c/n3gJtb/LU0Cl) |
| Count of COG0099 | Chromosomal unclonable gene | [(8)](https://paperpile.com/c/n3gJtb/LU0Cl) |
| Count of COG1314 | Chromosomal unclonable gene | [(8)](https://paperpile.com/c/n3gJtb/LU0Cl) |
| Count of COG0049 | Chromosomal unclonable gene | [(8)](https://paperpile.com/c/n3gJtb/LU0Cl) |
| Count of COG0336 | Chromosomal unclonable gene | [(8)](https://paperpile.com/c/n3gJtb/LU0Cl) |
| Count of COG3248 | Chromosomal unclonable gene | [(8)](https://paperpile.com/c/n3gJtb/LU0Cl) |
| Count of COG0185 | Chromosomal unclonable gene | [(8)](https://paperpile.com/c/n3gJtb/LU0Cl) |
| Count of COG0051 | Chromosomal unclonable gene | [(8)](https://paperpile.com/c/n3gJtb/LU0Cl) |
| Count of COG2814 | Chromosomal unclonable gene | [(8)](https://paperpile.com/c/n3gJtb/LU0Cl) |
| Count of COG1475 | Chromosomal unclonable gene | [(8)](https://paperpile.com/c/n3gJtb/LU0Cl) |
| Count of COG0227 | Chromosomal unclonable gene | [(8)](https://paperpile.com/c/n3gJtb/LU0Cl) |
| Count of COG0057 | Chromosomal unclonable gene | [(8)](https://paperpile.com/c/n3gJtb/LU0Cl) |
| Count of COG0501 | Chromosomal unclonable gene | [(8)](https://paperpile.com/c/n3gJtb/LU0Cl) |
| Count of COG4465 | Chromosomal unclonable gene | [(8)](https://paperpile.com/c/n3gJtb/LU0Cl) |
| Count of COG0534 | Chromosomal unclonable gene | [(8)](https://paperpile.com/c/n3gJtb/LU0Cl) |
| Count of COG0081 | Chromosomal unclonable gene | [(8)](https://paperpile.com/c/n3gJtb/LU0Cl) |
| Count of COG0335 | Chromosomal unclonable gene | [(8)](https://paperpile.com/c/n3gJtb/LU0Cl) |
| Count of COG0583 | Chromosomal unclonable gene | [(8)](https://paperpile.com/c/n3gJtb/LU0Cl) |
| Count of Hok | Plasmid genes, toxin-antixoin | [(9)](https://paperpile.com/c/n3gJtb/iOM3a) |
| Count of TisB | Plasmid genes, toxin-antixoin | [(9)](https://paperpile.com/c/n3gJtb/iOM3a) |
| Count of SymE | Plasmid genes, toxin-antixoin | [(9)](https://paperpile.com/c/n3gJtb/iOM3a) |
| Count of CcdB-CcdA | Plasmid genes, toxin-antixoin | [(9)](https://paperpile.com/c/n3gJtb/iOM3a) |
| Count of ParE-ParD | Plasmid genes, toxin-antixoin | [(9)](https://paperpile.com/c/n3gJtb/iOM3a) |
| Count of MazF-MazE | Plasmid genes, toxin-antixoin | [(9)](https://paperpile.com/c/n3gJtb/iOM3a) |
| Count of Kid-Kis | Plasmid genes, toxin-antixoin | [(9)](https://paperpile.com/c/n3gJtb/iOM3a) |
| Count of HicA-HicB | Plasmid genes, toxin-antixoin | [(9)](https://paperpile.com/c/n3gJtb/iOM3a) |
| Count of RelE-RelB | Plasmid genes, toxin-antixoin | [(9)](https://paperpile.com/c/n3gJtb/iOM3a) |
| Count of VapC-VapB | Plasmid genes, toxin-antixoin | [(9)](https://paperpile.com/c/n3gJtb/iOM3a) |
| Count of Doc-PhD | Plasmid genes, toxin-antixoin | [(9)](https://paperpile.com/c/n3gJtb/iOM3a) |
| Count of RatA-RatB | Plasmid genes, toxin-antixoin | [(9)](https://paperpile.com/c/n3gJtb/iOM3a) |
| Count of HipA-HipB | Plasmid genes, toxin-antixoin | [(9)](https://paperpile.com/c/n3gJtb/iOM3a) |
| Count of ToxN-ToxI | Plasmid genes, toxin-antixoin | [(9)](https://paperpile.com/c/n3gJtb/iOM3a) |
| Count of YeeV-Yeeu | Plasmid genes, toxin-antixoin | [(9)](https://paperpile.com/c/n3gJtb/iOM3a) |
| Count of CptA-CptB | Plasmid genes, toxin-antixoin | [(9)](https://paperpile.com/c/n3gJtb/iOM3a) |
| Count of GhoT-GhoS | Plasmid genes, toxin-antixoin | [(9)](https://paperpile.com/c/n3gJtb/iOM3a) |
| Count of Par gene | Plasmid gene | [(10)](https://paperpile.com/c/n3gJtb/rEpaP) |
| Count of Psi gene | Plasmid gene | [(11)](https://paperpile.com/c/n3gJtb/B5BhT) |
| Count of Tra gene | Plasmid gene, responsible for plasmid transfer | [(12)](https://paperpile.com/c/n3gJtb/nHKDW) |
| Count of Stb gene | Plasmid gene, responsible for plasmid stability | [(13)](https://paperpile.com/c/n3gJtb/Df1QX) |
| Count of Mob gene | Plasmid gene | [(14)](https://paperpile.com/c/n3gJtb/aLUyL) |
| Count of relaxase gene | Plasmid gene | [(15)](https://paperpile.com/c/n3gJtb/mqLPb) |
| Count of Type IV pili coupling protein | Plasmid gene | [(16)](https://paperpile.com/c/n3gJtb/dBTSd) |
| Count of VirB gene | Plasmid gene | [(16)](https://paperpile.com/c/n3gJtb/dBTSd) |

**Supplementary Table S2.** Plasmidic and chromosomal Pfam domains used in Deeplasmid classification.

**Supplementary Table S3.** We computed the AUC per each of the 12 models (2 models per fold) on ACLAME+PLSDB+Refseq training

| Fold | AUC |
| --- | --- |
| 0 | 0.988026 |
| 1 | 0.987749 |
| 2 | 0.98809 |
| 3 | 0.987104 |
| 4 | 0.985515 |
| 5 | 0.988614 |
| 0 | 0.986792 |
| 1 | 0.987262 |
| 2 | 0.988006 |
| 3 | 0.986709 |
| 4 | 0.986483 |
| 5 | 0.987637 |

### **Supplementary Table S4: AUC per k-fold and average for combinations of features.** The AUC achieved for various combinations of features on ACLAME+PLSDB+Refseq training shows the best AUC is achieved when keeping more features.

| **AUC** | **1** | **2** | **3** | **4** | **5** | **6** | **7** | **8** | **9** | **10** | **11** | **12** | **AVG** | **STD** |
| --- | --- | --- | --- | --- | --- | --- | --- | --- | --- | --- | --- | --- | --- | --- |
| All features (except_pfams) | 0.8889 | 0.8921 | 0.914 | 0.896 | 0.8974 | 0.9035 | 0.8898 | 0.8835 | 0.9124 | 0.8964 | 0.8962 | 0.8944 | **0.89705** | **0.002612** |
| All features_minus_aa_avg_len | 0.8989 | 0.8833 | 0.889 | 0.8818 | 0.8914 | 0.8933 | 0.8889 | 0.8684 | 0.905 | 0.8789 | 0.8888 | 0.9006 | **0.889025** | **0.002917** |
| All features_minus_len_sequence | 0.9105 | 0.8707 | 0.9163 | 0.9019 | 0.9038 | 0.8934 | 0.9056 | 0.8843 | 0.9174 | 0.8869 | 0.9064 | 0.8955 | **0.89939** | **0.003993** |
| All features_minus_gc_content | 0.9068 | 0.8916 | 0.9021 | 0.8779 | 0.8997 | 0.8962 | 0.8952 | 0.8903 | 0.9133 | 0.877 | 0.9028 | 0.8924 | **0.89544** | **0.003093** |
| hits_chrom_plasmid_proteins_ORIs_only | 0.8561 | 0.8529 | 0.8593 | 0.8497 | 0.863 | 0.8544 | 0.8473 | 0.8445 | 0.8637 | 0.8505 | 0.857 | 0.8595 | **0.854825** | **0.001753** |
| All features_minus_hits_chrom_plasmid_proteins_ORIs | 0.8574 | 0.8291 | 0.8411 | 0.8516 | 0.8251 | 0.8557 | 0.8609 | 0.8381 | 0.8542 | 0.8517 | 0.844 | 0.8613 | **0.84751** | **0.003477** |
| All features_minus_gene_count | 0.889 | 0.8789 | 0.9122 | 0.8803 | 0.9057 | 0.8974 | 0.9007 | 0.8786 | 0.9143 | 0.8806 | 0.8952 | 0.891 | **0.89365** | **0.003695** |
| gene_count_aa_avg_len_only | 0.8124 | 0.7841 |  | 0.7837 | 0.7841 | 0.8018 | 0.8104 | 0.7679 | 0.821 | 0.7901 | 0.7746 | 0.7771 | **0.79156** | **0.004993** |
| All features_minus_homopolymer | 0.8835 | 0.8735 | 0.9143 | 0.8911 | 0.8967 | 0.8862 | 0.881 | 0.8698 | 0.9088 | 0.8878 | 0.896 | 0.8925 | **0.8901** | **0.003748** |
| gc_content_lensequence_only | 0.7434 | 0.7175 | 0.7439 | 0.7178 | 0.7163 | 0.7497 | 0.7425 | 0.7237 | 0.7324 | 0.7328 | 0.7204 | 0.7406 | **0.73175** | **0.003515** |

### Code Repository

The GitHub code repository is at: <https://github.com/wandreopoulos/deeplasmid>

This repo is mainly intended to store code for the paper and open-source community access.

The classifier/dl directory had the cleaned up code. You may delete or ignore other dirs, like bin and binning dirs.

The **docker** branch contains the docker-specific code that works on *Mac (with docker).* The **master** branch is the version that can be deployed natively on a Linux system.

### Software Design

Supplementary Figures S1-S2 show a flowchart of the directory structure with files input to prediction and output from training and the software design with the main modules.

### The training and test datasets

The training (ACLAME+PLSDB+refseq) and test datasets (IMG) are accessible under: <https://portal.nersc.gov/dna/microbial/assembly/deeplasmid/>

### **Supplementary Figures**


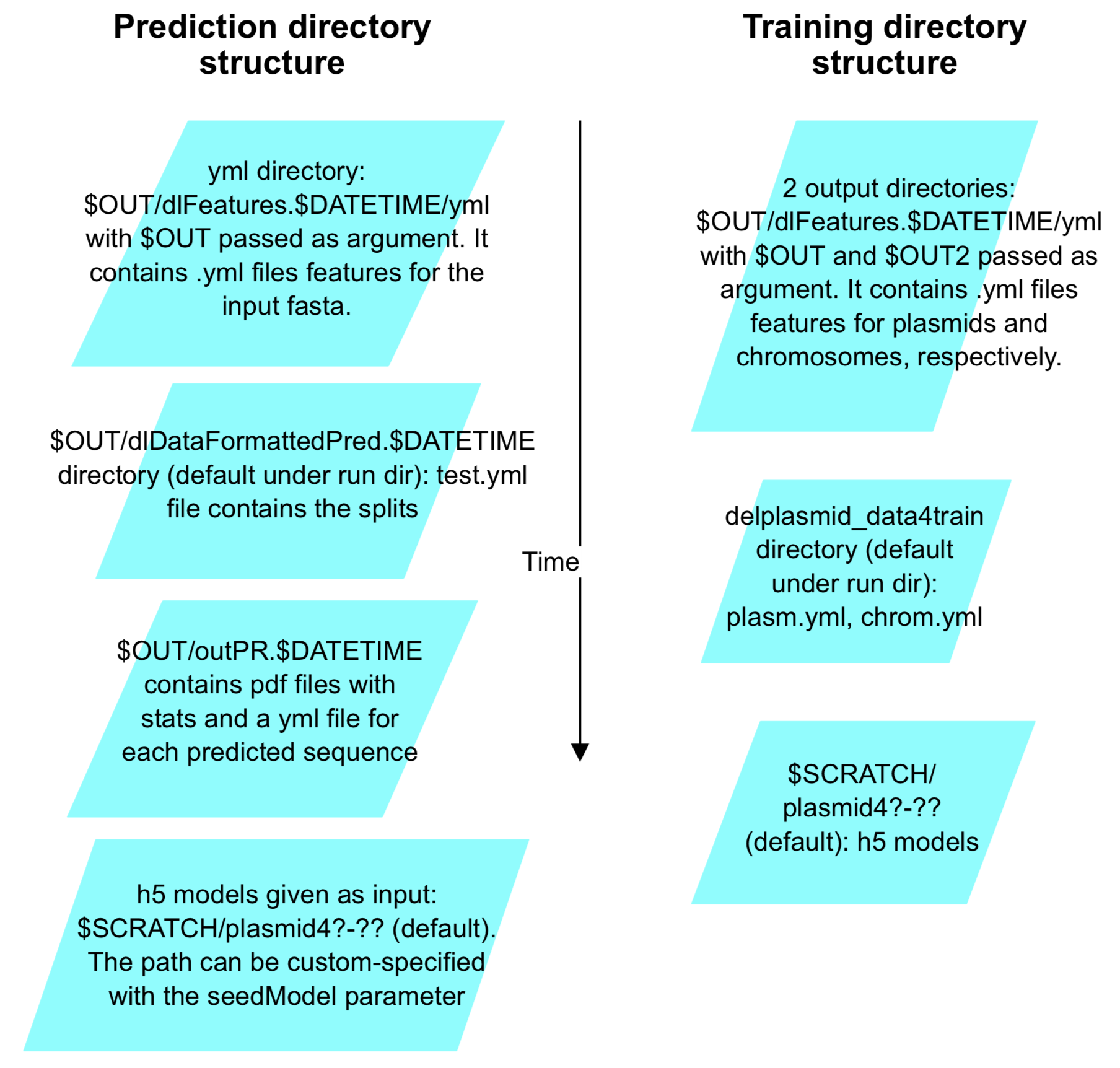


**Supplementary Figure S1:** The directory structure expected for input to the training and prediction and the output directories with files produced. See README file under codebase for examples of running training and prediction with the deeplasmid tool.


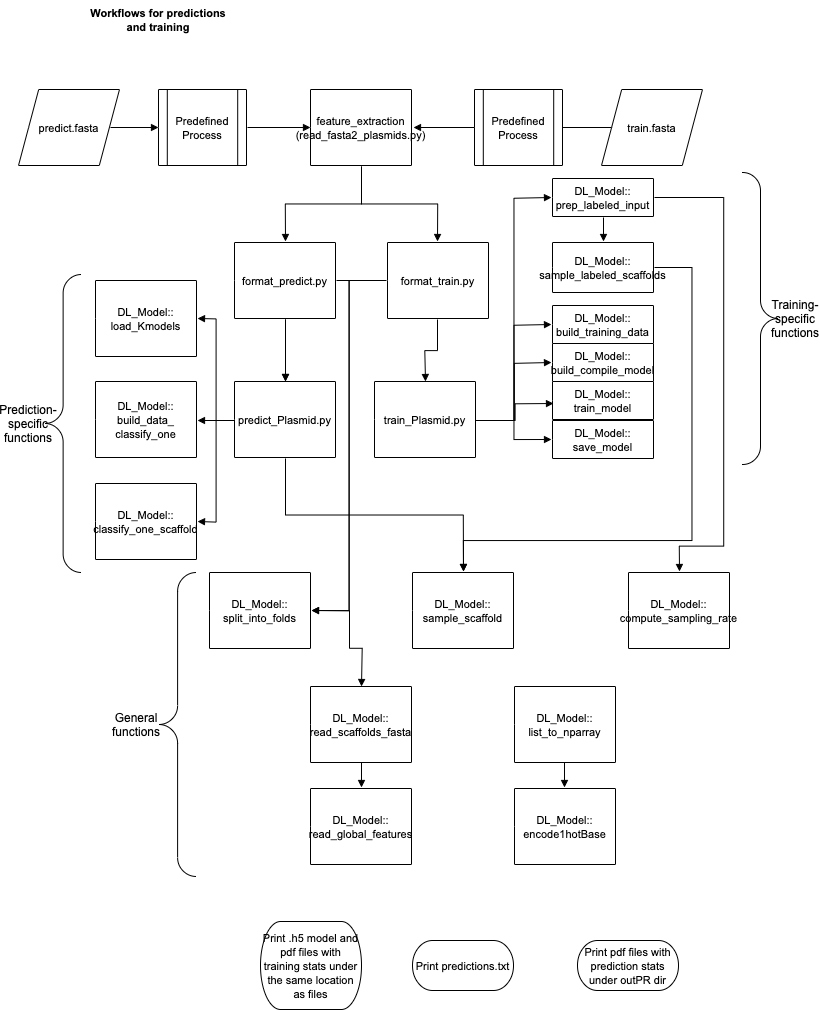


**Supplementary Figure S2:** The design of the codebase for prediction and training with the main classes, important functions, and call graph.

### Deep learning model architecture


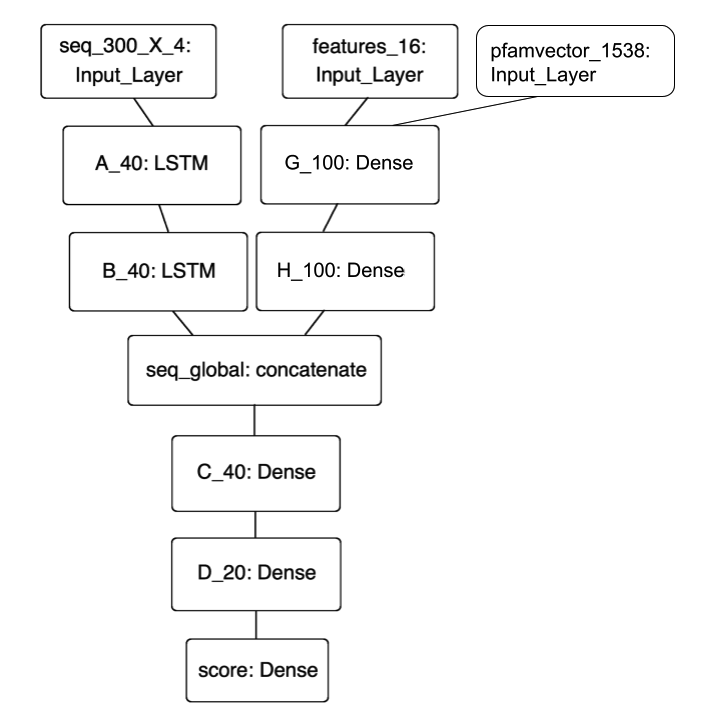


**Supplementary Figure S3: Model topology of Deeplasmid Neural Network.** Fully connected layers are denoted as ’Dense’. The numbers in the boxes are the sizes of the output features. A dropout rate of 0.1 is applied in-between all layers. Dropout sets weights to zero in order to prevent overfitting and allow the training to generalize to new datasets.


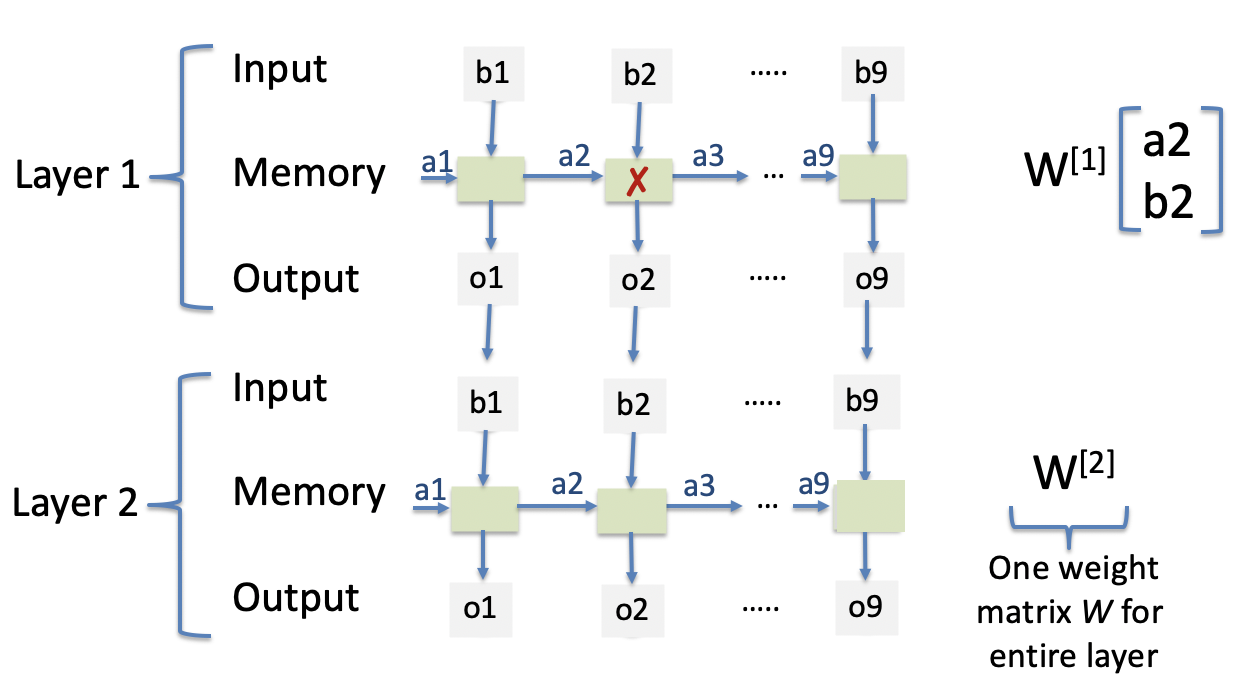


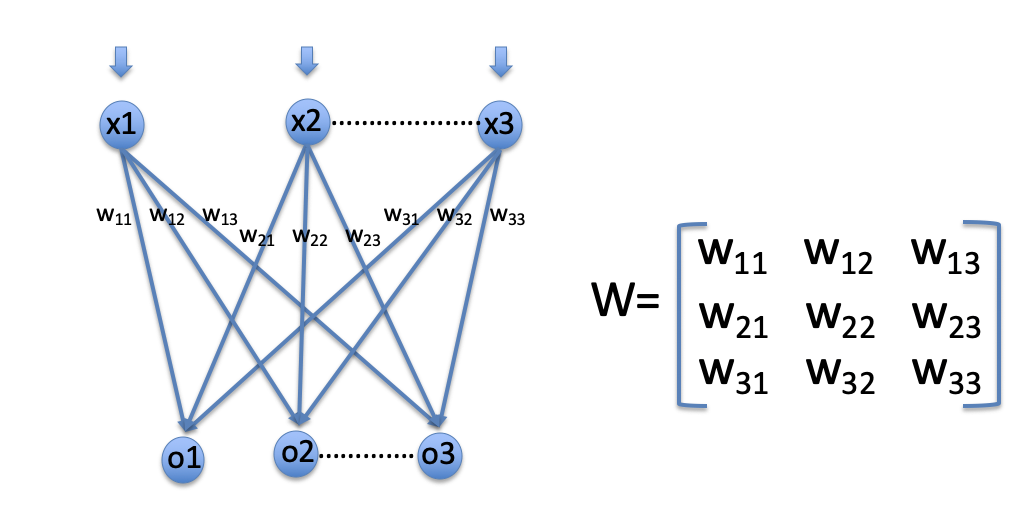


**Supplementary Figure S4:** A layered LSTM architecture (top), and a basic dense-layer architecture (bottom).

### Difference of features between chromosomal DNA and plasmids


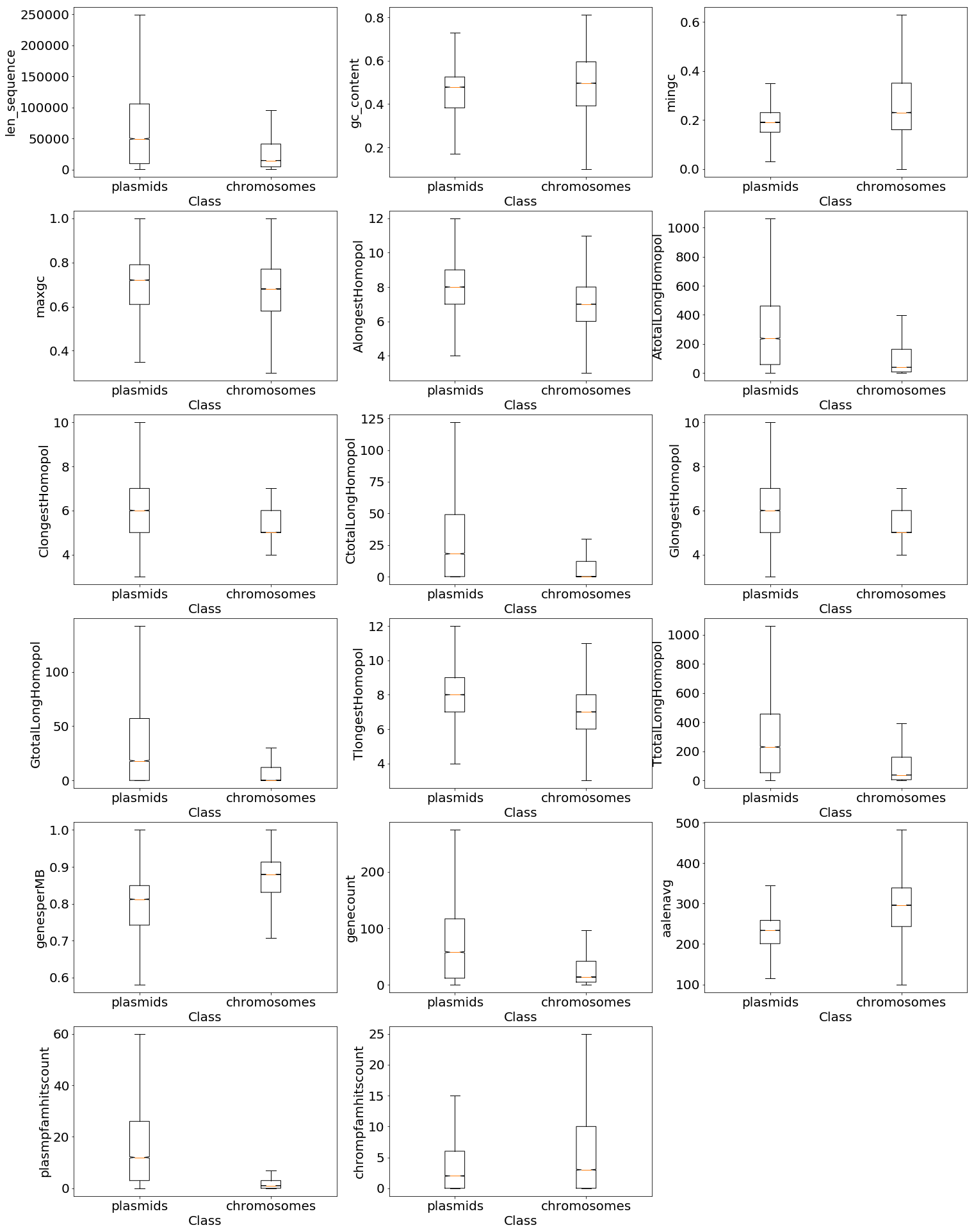


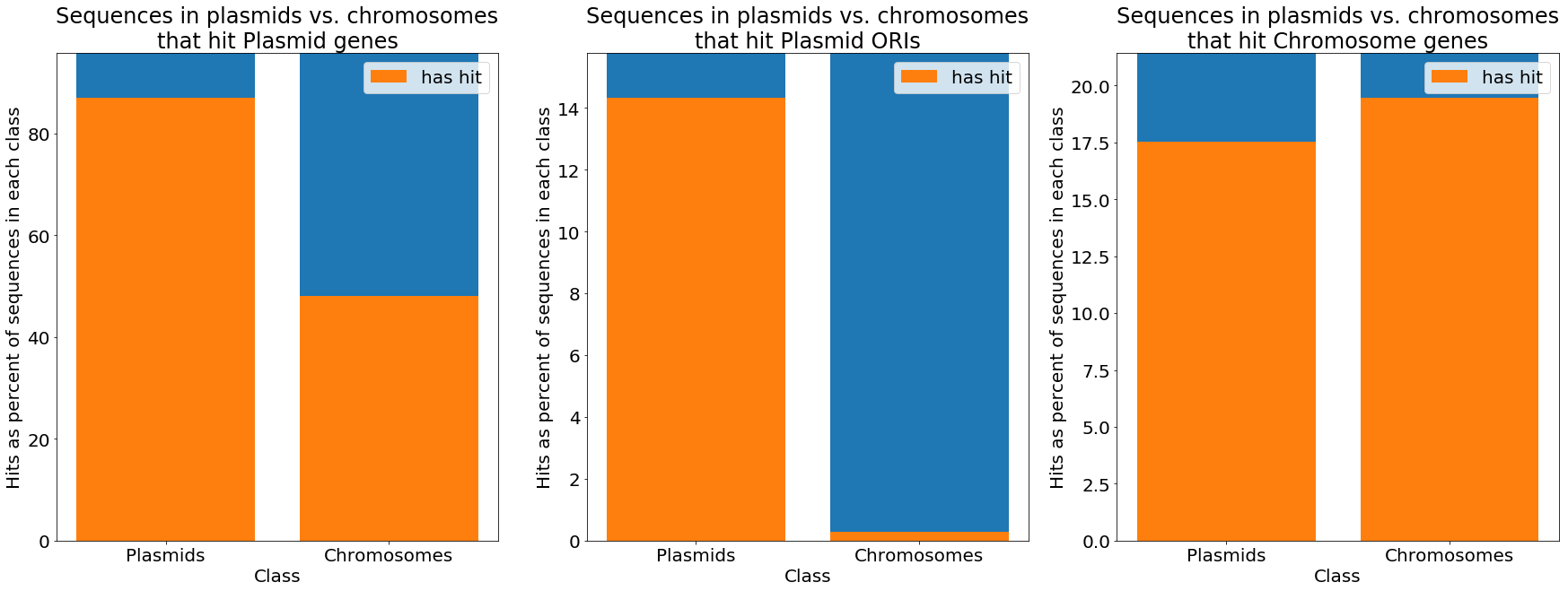


**Supplementary Figure S5:** The plasmid genes, chromosome genes, and plasmid ORI feature values compared between the two classes of chromosomal vs. plasmid on the ACLAME+PLSDB+refseq training dataset with 62,900 sequences of length 1K-330K bases

### Assembly of *Yersinia* circular plasmid


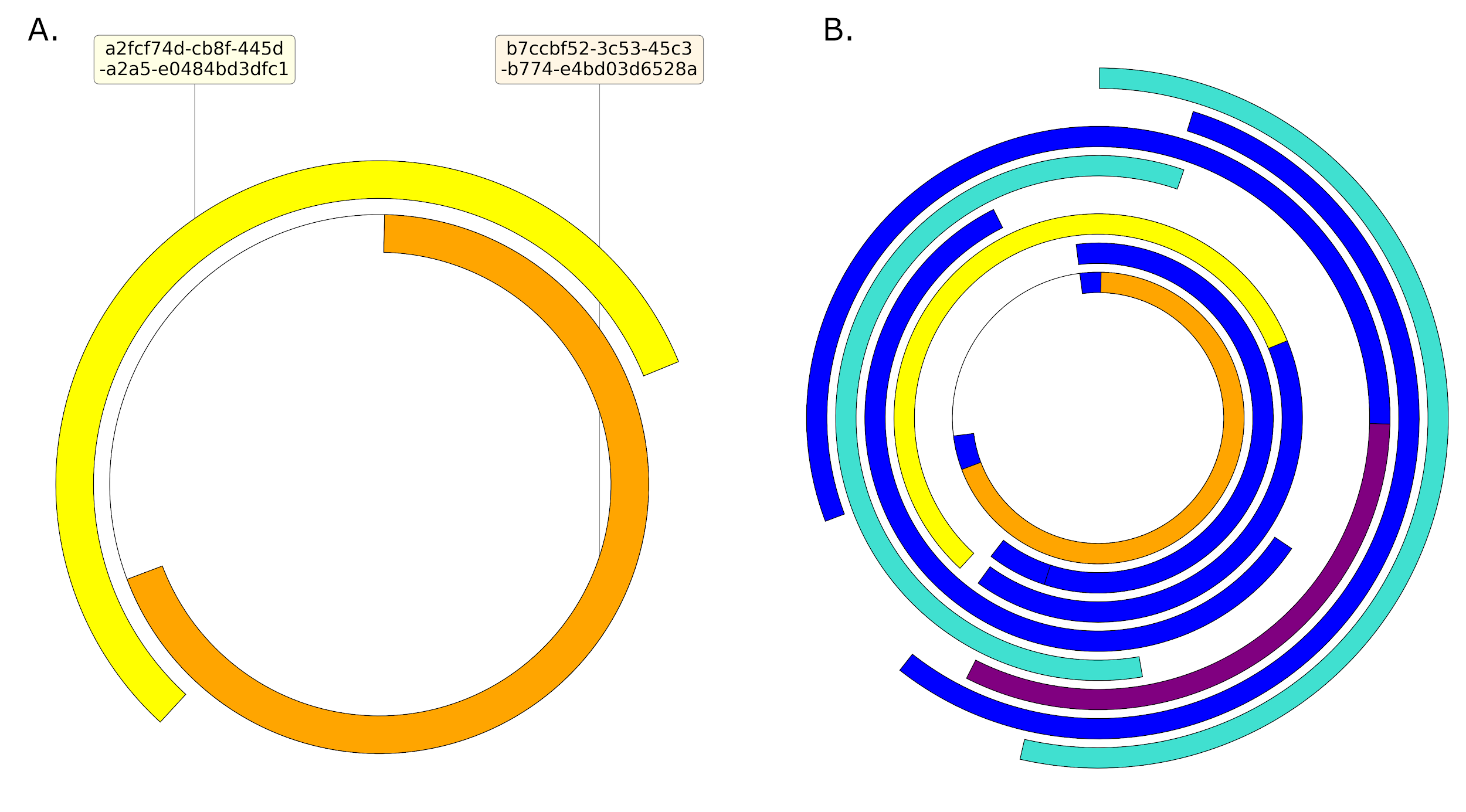


**Supplementary Figure S6:** Oxford Nanopore reads mapped onto novel plasmid assembly. Minimap2 was used to map fastq reads to the novel plasmid, which shows overlaps indicative of circular sequences. (A) Representative overlapping fastq reads. Read fastq headers are listed. (B) All fastq reads mapping to the plasmid sequence, with yellow and orange corresponding to the reads (A). Other colors in (B) are randomly assigned.

### References

[1. Rocha,E.P.C. and Danchin,A. (2002) Base composition bias might result from competition for metabolic resources. *Trends Genet.*, **18**, 291–294.](http://paperpile.com/b/n3gJtb/cZyT)

[2. Mei,J., Benashski,S. and Firshein,W. (1995) Interactions of the origin of replication (oriV) and initiation proteins (TrfA) of plasmid RK2 with submembrane domains of Escherichia coli. *J. Bacteriol.*, **177**, 6766–6772.](http://paperpile.com/b/n3gJtb/3f9r4)

[3. Fang,F.C. and Helinski,D.R. (1991) Broad-host-range properties of plasmid RK2: importance of overlapping genes encoding the plasmid replication initiation protein TrfA. *J. Bacteriol.*, **173**, 5861–5868.](http://paperpile.com/b/n3gJtb/Xw1hw)

[4. Kimelman,A., Levy,A., Sberro,H., Kidron,S., Leavitt,A., Amitai,G., Yoder-Himes,D.R., Wurtzel,O., Zhu,Y., Rubin,E.M., *et al.* (2012) A vast collection of microbial genes that are toxic to bacteria. *Genome Res.*, **22**, 802–809.](http://paperpile.com/b/n3gJtb/B9oBE)

[5. Light,J. and Molin,S. (1983) Post-transcriptional control of expression of the repA gene of plasmid R1 mediated by a small RNA molecule. *EMBO J.*, **2**, 93–98.](http://paperpile.com/b/n3gJtb/0aoMX)

[6. Grazziotin,A.L., Vidal,N.M. and Venancio,T.M. (2015) Uncovering major genomic features of essential genes in Bacteria and a methanogenic Archaea. *FEBS J.*, **282**, 3395–3411.](http://paperpile.com/b/n3gJtb/VE4Tm)

[7. Tazzyman,S.J. and Bonhoeffer,S. (2015) Why There Are No Essential Genes on Plasmids. *Mol. Biol. Evol.*, **32**, 3079–3088.](http://paperpile.com/b/n3gJtb/1Pjom)

[8. Sorek,R., Zhu,Y., Creevey,C.J., Francino,M.P., Bork,P. and Rubin,E.M. (2007) Genome-wide experimental determination of barriers to horizontal gene transfer. *Science*, **318**, 1449–1452.](http://paperpile.com/b/n3gJtb/LU0Cl)

[9. Unterholzner,S.J., Poppenberger,B. and Rozhon,W. (2013) Toxin-antitoxin systems: Biology, identification, and application. *Mob. Genet. Elements*, **3**, e26219.](http://paperpile.com/b/n3gJtb/iOM3a)

[10. Gerdes,K., Møller-Jensen,J. and Bugge Jensen,R. (2000) Plasmid and chromosome partitioning: surprises from phylogeny. *Mol. Microbiol.*, **37**, 455–466.](http://paperpile.com/b/n3gJtb/rEpaP)

[11. Petrova,V., Chitteni-Pattu,S., Drees,J.C., Inman,R.B. and Cox,M.M. (2009) An SOS inhibitor that binds to free RecA protein: the PsiB protein. *Mol. Cell*, **36**, 121–130.](http://paperpile.com/b/n3gJtb/B5BhT)

[12. Zatyka,M. and Thomas,C.M. (1998) Control of genes for conjugative transfer of plasmids and other mobile elements. *FEMS Microbiol. Rev.*, **21**, 291–319.](http://paperpile.com/b/n3gJtb/nHKDW)

[13. Guynet,C., Cuevas,A., Moncalián,G. and de la Cruz,F. (2011) The stb operon balances the requirements for vegetative stability and conjugative transfer of plasmid R388. *PLoS Genet.*, **7**, e1002073.](http://paperpile.com/b/n3gJtb/Df1QX)

[14. Wang,P., Zhu,Y., Zhang,Y., Zhang,C., Xu,J., Deng,Y., Peng,D., Ruan,L. and Sun,M. (2016) Mob/oriT, a mobilizable site-specific recombination system for unmarked genetic manipulation in Bacillus thuringiensis and Bacillus cereus. *Microb. Cell Fact.*, **15**, 108.](http://paperpile.com/b/n3gJtb/aLUyL)

[15. Garcillán-Barcia,M.P., Francia,M.V. and de la Cruz,F. (2009) The diversity of conjugative relaxases and its application in plasmid classification. *FEMS Microbiol. Rev.*, **33**, 657–687.](http://paperpile.com/b/n3gJtb/mqLPb)

[16. Smillie,C., Garcillán-Barcia,M.P., Francia,M.V., Rocha,E.P.C. and de la Cruz,F. (2010) Mobility of plasmids. *Microbiol. Mol. Biol. Rev.*, **74**, 434–452.](http://paperpile.com/b/n3gJtb/dBTSd)
